# Autoinflammatory Keratinization Diseases—The Concept, Pathophysiology, and Clinical Implications

**DOI:** 10.1007/s12016-023-08971-3

**Published:** 2023-12-16

**Authors:** Leszek Blicharz, Joanna Czuwara, Lidia Rudnicka, Antonio Torrelo

**Affiliations:** 1 Department of Dermatology, Medical University of Warsaw, 02-008 Warsaw, Poland; 2 Department of Dermatology, University Children’s Hospital Niño Jesús, 28009 Madrid, Spain

**Keywords:** Autoinflammation, Autoinflammatory keratinization diseases, *CARD14*, *IL36RN*, NADED, *NLRP1*

## Abstract

Recent advances in medical genetics elucidated the background of diseases characterized by superficial dermal and epidermal inflammation with resultant aberrant keratosis. This led to introducing the term autoinflammatory keratinization diseases encompassing entities in which monogenic mutations cause spontaneous activation of the innate immunity and subsequent disruption of the keratinization process. Originally, autoinflammatory keratinization diseases were attributed to pathogenic variants of *CARD14* (generalized pustular psoriasis with concomitant psoriasis vulgaris, palmoplantar pustulosis, type V pityriasis rubra pilaris), *IL36RN* (generalized pustular psoriasis without concomitant psoriasis vulgaris, impetigo herpetiformis, acrodermatitis continua of Hallopeau), *NLRP1* (familial forms of keratosis lichenoides chronica), and genes of the mevalonate pathway, i.e., *MVK*, *PMVK*, *MVD*, and *FDPS* (porokeratosis). Since then, endotypes underlying novel entities matching the concept of autoinflammatory keratinization diseases have been discovered (mutations of *JAK1*, *POMP*, and *EGFR*)*.* This review describes the concept and pathophysiology of autoinflammatory keratinization diseases and outlines the characteristic clinical features of the associated entities. Furthermore, a novel term for NLRP1-associated autoinflammatory disease with epithelial dyskeratosis (NADED) describing the spectrum of autoinflammatory keratinization diseases secondary to *NLRP1* mutations is proposed.

## Introduction

In 1997, pathogenic variants in the gene *MEFV* causing familial Mediterranean fever were identified [1]. This discovery marked a breakthrough in the understanding of systemic diseases caused by congenital hyperactivation of the innate immune system that manifests by heterogenous patterns of systemic inflammation [2, 3]. Later, more disorders sharing similar pathogenetic pathways resulting in self-limited episodes of fever and serosal, synovial, and cutaneous symptoms were genetically characterized [4]. This emerging group was named “autoinflammatory diseases.” The phenomenon of autoinflammation was distinguished from autoimmunity by lacking the typical stigmata of the latter, e.g., high-titer autoantibodies or antigen-specific T lymphocytes.

Two decades after explaining the cause of familial Mediterranean fever, Akiyama et al. proposed the term “autoinflammatory keratinization diseases” (AiKDs) for a subcategory of autoinflammatory disorders characterized by superficial dermal and epidermal inflammation altering the keratinization process [5]. The hallmarks of AiKDs involve the hyperactivation of the innate immune system caused primarily by genetic factors and the resulting mixed pathomechanisms of autoinflammation and autoimmunity.

The purpose of this review is to present the concept and pathophysiology of AiKDs and outline the characteristic features of the conditions included in this group.

## Innate Immunity and Autoinflammation

The innate immune system is a conservative line of defense preventing loss of homeostasis induced by environmental and endogenous stressors [6]. Its primary role is to control the breach by infectious agents, but its function is being constantly elucidated in heterogenous physiological processes [7]. Innate immunity comprises constitutive and inducible mechanisms [8]. Constitutive immune responses involve restriction factors, antimicrobial peptides, basal autophagy, and proteasomal degradation. Inducible responses are dependent on sensing by pattern recognition receptors (PRRs), e.g., toll-like receptors (TLRs) and nucleotide-binding oligomerization domain (NOD)-like receptors (NLR) showing high affinity toward conserved microbial structures [9]. PRRs sense pathogen-associated molecular patterns (PAMPs) and danger-associated molecular patterns (DAMPs). Subsequent downstream signaling from PRRs elicits the production of cytokines and other molecular signals orchestrating the organism’s response to the detected perturbation. Among others, these processes also influence the fine-tuning of acquired immune responses, which can result in the simultaneous activation of mixed pathways of autoimmunity and autoinflammation [10].

### Pathogenesis of Autoinflammatory Diseases

Genetically determined malfunctioning of innate immunity can cause systemic inflammation to develop spontaneously or upon a minor trigger [11]. This can be caused by either loss-of-function mutations in genes responsible for suppressing the inflammatory responses or gain-of-function mutations in genes that propagate these processes [12].

Theoretically, every modality of innate immune response may be affected. However, the most uniform classification of autoinflammatory syndromes based on the underlying pathophysiological mechanisms distinguishes four primary groups of entities [12, 13]:

1. Inflammasomopathies and other disorders associated with aberrant IL-1 family signaling
2. Type I interferonopathies
3. Disorders of NF-κB and/or aberrant TNF activity
4. Diseases caused by other miscellaneous mechanisms

#### Inflammasomopathies and Other Disorders Associated with Aberrant IL-1 Family Signaling

IL-1 cytokine superfamily (i.e., IL-1α, IL-1β, IL-18, and IL-36) is a primary factor orchestrating inflammatory reactions in response to tissue damage [14]. The active forms of IL-1β and IL-18 are generated from their inactive precursors by caspase-1 proteolysis. The latter is an enzyme activated by inflammasomes, i.e., protein complexes assembling upon conformational changes in core nucleating proteins induced by cellular stressors [15–17]. Various inflammasomes, e.g., pyrin, NLRP1, NRLP3, NLRP12, and NLRC4, have been distinguished based on the associated nucleating proteins. Importantly, the cellular expression of inflammasomes and their substrates varies in different tissues; hence, their spontaneous activation in autoinflammatory disorders may be associated with organ-specific symptoms [16, 18]. Considering the primary role of inflammasomes in triggering IL-1-mediated responses, inflammasomopathies are discussed together with disorders associated with aberrant IL-1-dependent signaling. IL-36, a member of the IL-1 superfamily highly expressed in keratinocytes and endothelial cells, shows a different mode of activation dependent primarily on soluble neutrophil proteases, such as cathepsin G [19].

#### Interferonopathies

Interferons are cytokines involved in innate and adaptive immune responses [20]. They exert their action through type I and II receptors with subsequent signal transduction through Janus kinases [21]. Three groups of interferons have been distinguished: type I (IFNα, IFNβ signaling through the type I IFN receptor), type II (IFNγ signaling through type II IFN receptor), and type III (IFNλ signaling through a receptor sharing the same pathways of downstream signaling with type I IFN) [22–24]. Described autoinflammatory syndromes are due to type I IFN abnormalities [25, 26]. This group of cytokines is primarily associated with antiviral responses dependent on the sensing of viral DNA or RNA. Therefore, the underlying pathomechanisms of interferonopathies involve improper sensing or accumulation of nucleic acids or waste proteins (e.g., due to proteasomal abnormalities) and amplified receptor signaling [26].

#### Disorders of NF-κB and/or Aberrant TNF Activity

The NF-κB complex mediates downstream signaling triggered by both intra- and extracellular danger signals [27]. The result of NF-κB complex activation is the release of transcription factors enhancing the expression of proinflammatory molecules, with the TNF cytokine family as its primary effector and reciprocal regulator [27, 28]. Among the many processes which can lead to the hyperactivation of the NF-κB cascade and TNF function are decreased activity of the NF-κB negative regulators (e.g., A20 haploinsufficiency), increased activation by factors such as caspase recruitment domain–containing protein (CARD), and activating mutations in genes encoding TNF receptor 1 (e.g., in TRAPS syndrome) [29–31].

#### Diseases Caused by Other Miscellaneous Mechanisms

Novel discoveries regarding the innate immune system explain the pathogenesis of autoinflammatory syndromes that do not fall into the described categories. Among those newly identified pathomechanisms are hyperreactive external calcium entry in B cells and expansion of innate inflammatory cells seen in PLAID syndrome, disruption of transport from the Golgi apparatus to the endoplasmic reticulum due to defective coatomer protein subunit α in COPA syndrome, and impairment of pathways regulating actin polarization and cytoskeletal architecture in CDC42 deficiency [32–34]. Possibly, identification of new pathomechanisms could augment new categories of autoinflammatory diseases with the resultant discerning of more well-defined subgroups sharing similar pathways.

### Autoinflammation and Autoimmunity

Autoimmunity is a state of disrupted acquired immune response in which T cells and B cells are primary effectors [35]. The hallmark of autoimmunity involves improper sensing of autoantigens as danger signals with the resultant formation of autoantibodies targeting functional structures of the cell such as the nucleus [36].

Recently, innate immune system has been revealed as a significant contributing factor to the initiation and amplification of autoimmune diseases [26, 37–39].

In the initiation phase, autoantigens are detected and internalized by antigen-presenting cells in a TLR-dependent manner. This causes the activation of caspase-1 and inflammasome-induced production of active IL-1 cytokine family members [40]. Subsequent signaling through the IL-1 receptor (IL-1R) promotes the survival and differentiation of naïve T cells which induce B cells to start antibody production [41]. Additionally, a stable differentiation of Th17 cells from naïve T cells also depends on the IL-1 family cytokines (mainly IL-1α and IL-1β).

TLR-dependent signaling further elicits the production of IFN-α stimulating the cascade of dendritic cell maturation, presentation of autoantigens, and lymphocyte recruitment with subsequent production of autoantibodies [35]. IFN-α is also secreted by plasmacytoid dendritic cells upon internalization of autoantigen-autoantibody immune complexes which activate other dendritic cells and T cells [42, 43]. This promotes a self-sustained amplification of inflammation.

Another example of the close relationship between autoinflammation and autoimmunity is the CARD-dependent NOD-2 activation, which leads both to the activation of IL-1β, mediated by caspase 1, and NF-κB-induced transcription of proinflammatory factors [44].

It is therefore clear that some diseases considered mainly autoinflammatory can also be associated with simultaneous activation of adaptive immunity and autoimmune stigmata. However, in contrast to the monogenic background of most autoinflammatory diseases, autoimmune disorders are more frequently associated with polygenic inheritance, with certain susceptibility loci being attributed to the pathways listed above [45, 46].

## Autoinflammatory Keratinization Diseases

Genetic susceptibility for the development of inflammatory keratinization diseases, e.g., psoriasis, is well established [47]. The familial predetermination has a primarily polygenic background. In these cases, the pathogenesis is thought to be largely driven by the adaptive immunity. The discovery of monogenic aberrations causing hyperactivation of the innate immune system led to developing the umbrella term AiKDs to reflect the different etiology and clinical implications of inflammatory keratinization diseases with mixed pathomechanisms of autoinflammation and autoimmunity [48]. According to Akiyama et al. AiKDs are defined by the following criteria [5]:

1. The inflammation is primarily confined to the epidermis and upper dermis;
2. The inflammation leads to hyperkeratosis constituting the main characteristic phenotype of AiKDs;
3. AiKDs develop mainly due to genetic causative factors associated with the hyperactivation of innate immunity;
4. The concept of AiKDs encompasses diseases with mixed pathomechanisms of autoinflammation and autoimmunity.

### The Autoinflammatory Keratinization Disease Spectrum

Originally, disorders included in the spectrum of AiKDs involved variants of generalized pustular psoriasis (GPP), palmoplantar pustulosis, type V (atypical juvenile) pityriasis rubra pilaris (PRP), impetigo herpetiformis, acrodermatitis continua, and familial forms of keratosis lichenoides chronica [5]. Detection of new pathogenetic mechanisms dependent on the hyperactivation of innate immunity is causing the spectrum of AiKDs to expand [49]. Since the introduction of this concept, certain changes in nomenclature have also been proposed to better categorize AiKDs based on the underlying pathomechanism. For example, a compound term of *CARD14*-associated papulosquamous eruption (CAPE) was coined for cases of psoriasis and PRP sharing similar clinical features (age of onset, location, family history) and favorable treatment outcomes dependent on IL-12/IL-23 blockade [50].

Described AiKDs fall into the following categories of autoinflammatory diseases described above:

1. Inflammasomopathies and other disorders associated with aberrant IL-1 family signaling due to
  - Deficiency of the IL-36 receptor antagonist (IL-36Ra) (DITRA)
  - Deficiency of the IL-1 receptor antagonist (IL-1Ra) (DIRA)
  - NLRP1 hyperactivation
2. Disorders of NF-κB and/or aberrant TNF activity due to
  - CARD14 hyperactivation
  - Adaptor protein complex 1 subunit σ1C (AP1S3) deficiency
3. Diseases caused by other miscellaneous mechanisms
  - Mevalonate pathway abnormalities
  - Janus kinase 1 hyperactivity
  - Proteasome maturation protein deficiency
  - Epidermal growth factor receptor deficiency

In some reviews, hidradenitis suppurativa is included within the AiKDs [51, 52]. However, due to the complex and most often polygenic background of this disease, incompatibility of the clinical features, and the presence of deep inflammatory infiltrates in histopathology, this article will not discuss hidradenitis suppurativa.

**Fig. 1:**
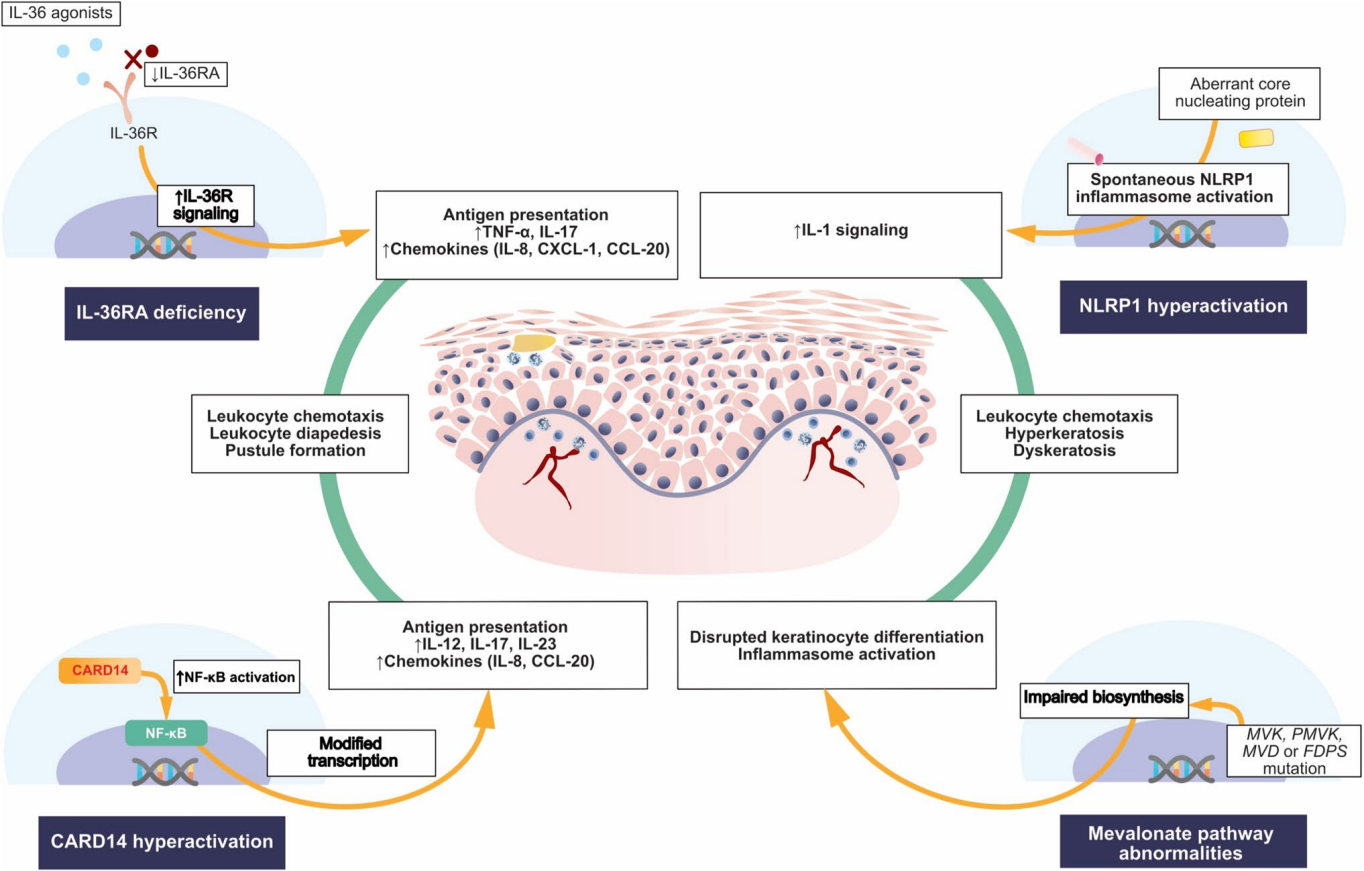
Pathogenesis of the most significant autoimmune keratinization diseases associated with (1) increased IL-36R signaling caused by deficiency of IL-36RA, (2) CARD14 hyperactivation causing the upregulation of NF-κB, (3) NLRP1 inflammasome activation, and (4) mevalonate pathway abnormalities causing impaired synthesis of isoprenoids. CARD14—caspase recruitment domain family member 14; CCL-20—chemokine C-C motif ligand 20; FDPS—farnesyl diphosphate synthase; IL—interleukin; CXCL-1—chemokine C-X-C motif ligand 1; IL-36R—interleukin 36 receptor; IL-36RA—interleukin 36 receptor antagonist; MVD—mevalonate decarboxylase; MVK—mevalonate kinase; NF-κB—nuclear factor kappa-light-chain-enhancer of activated B cells; NLRP1—nucleotide-binding oligomerization domain-like receptor containing a PYRIN domain 1; PMVK—phosphomevalonate kinase; TNF—tumor necrosis factor

The pathogenesis of the most relevant AiKDs is illustrated in Fig. 1.

#### Inflammasomopathies and Other Disorders Associated with Aberrant IL-1 Family Signaling

##### IL-36Ra Deficiency

IL-36 family is composed of three agonists (IL-36α, IL-36β, and IL-36γ) and an antagonist (IL-36Ra) [53, 54]. IL-36 cytokines are primarily expressed by T cells, keratinocytes, and other cutaneous cells [55]. They promote inflammation by stimulating antigen presentation and induction of immunocompetent cells.

Inactive full-length IL-36 is processed by enzymes derived from neutrophils, such as the cathepsin G (Cat G), elastase, and proteinase-3 [56]. This causes the upregulation of IL-36R signaling which triggers a cascade of proinflammatory factor release. In keratinocytes, activated IL-36β induces the upregulation of TNF, Th17 cytokines (IL-17A, IL-17C, IL-22), and chemokines (IL-8, chemokine [C-X-C motif] ligand 1 [CXCL-1], chemokine [C-C motif] ligand 20 [CCL-20]) [54]. This constitutes a classic psoriatic cytokine milieu and explains the causative role of IL-36 in driving the superficial cutaneous inflammation. Apart from causing chemotaxis of granulocytes, IL-36 also induces their adhesion and diapedesis [19, 57]. This observation is possibly the main reason for the occurrence of pustules in diseases associated with IL-36 signaling.

The negative regulation of IL-36 is mediated by IL-36Ra, whose active form is induced by neutrophilic elastase [58, 59]. Thus, deficiency of IL-36Ra caused by *IL36RN* loss-of-function mutations propagates IL-36-dependent signaling and the ensuing immune cascade described above.

In 2011, the role of *IL36RN* mutation resulting in IL-36Ra deficiency was first associated with a recessively inherited familial variant of generalized pustular psoriasis in 9 Tunisian families [60]. This led to propose a novel autoinflammatory disease referred to as DITRA (OMIM no. 605507). The same pathway was also demonstrated to underlie several sporadic GPP cases across the globe [61, 62]. Data analysis revealed that patients with IL-36Ra deficiency are typically characterized by early-onset systemic inflammation and the absence of concurrent psoriasis vulgaris (Fig. 6) [63]. Therefore, screening for *IL36RN* mutation is advisable in individuals presenting these clinical features. *IL36RN* mutations have also been detected in other pustular dermatoses, including acrodermatitis continua and impetigo herpetiformis [64, 65].

Among the histological features of diseases mediated by *IL36RN* mutations, the presence of hyperkeratosis may be elusive and overwhelmed by the massive neutrophilic infiltration [66]. This could raise the question of whether this subgroup should be included in the AiKD spectrum. However, the common view is that the processes of keratinocyte differentiation and proliferation are affected in these entities, which justifies their recognition as AiKDs.

##### IL-1Ra Deficiency

IL-1Ra deficiency (DIRA, OMIM no. 612852) follows the same pathogenetic concepts as the IL-36Ra deficiency (DITRA) and is based on impaired negative regulation of IL-1 cytokine superfamily [67, 68]. However, the broader physiological role of IL-1 cytokines and their more ubiquitous expression in different tissues translate to the involvement of multiple organs, including the skin, bones, and central nervous system [68, 69]. Excessive signaling via the IL-1R induces neutrophil chemotaxis, infiltration, and pustule formation [70]. Cutaneous symptoms resemble those in IL-36Ra deficiency and imitate GPP [67]. However, patients are more commonly characterized by a very early onset of autoinflammatory stigmata, severe systemic symptoms, and markedly elevated inflammatory markers.

To date, DIRA has not been proposed to fall into the spectrum of AiKDs, possibly due to the associated severe extracutaneous manifestations. However, the evident autoinflammatory etiology and cutaneous findings resulting from superficial inflammation substantiate the designation of DIRA as an AiKD.

##### NLRP1 Hyperactivation

NLRP1 is a core protein forming a part of an inflammasome complex [18, 71]. It contains five different domains: an aminoterminal pyrin domain (PYD), a NACHT domain, six leucine-rich repeat (LRR) domains, a function-to-find domain (FIIND), and a carboxyterminal CARD. NLRP1 is considered a primary sensor of danger signals in the epithelia, and its expression is particularly prominent in keratinocytes [72]. Gain-of-function mutations in the *NLRP1* gene cause excessive activation of the associated inflammasome with the resultant overproduction of the IL-1 cytokine superfamily causing pyroptosis and cell death [73]. Other mediators triggered by NLRP1 activation involve TNF, IL-5, IL-6, IL-8, IL-17, S100A9, and FGF7 which play an important role in altering keratinocyte differentiation and proliferation [74–76]. As a long-range effect, NLRP1-mediated inflammation may contribute to the acquisition of mutations with oncogenic potential [18]. NLRP1 hyperactivation was discovered in the prototypic AiKD, i.e., familial keratosis lichenoides chronica [71]. The latter is associated with germline gain-of-function mutations in PYD and LRR domains of NLRP1. However, more cases of NLRP1 dysregulation resulting in epithelial inflammation have been discovered, justifying the recognition of a novel, joint spectrum of AiKDs (see the “Nucleotide-Binding Oligomerization Domain-Like Receptor Containing a PYRIN Domain 1 (NLRP1)-Associated Autoinflammatory Disease With Epithelial Dyskeratosis (NADED)” section).

#### Disorders of NF-κB and/or Aberrant TNF Activity

##### CARD14 Hyperactivation

CARD14 regulates the central hub of intracellular signaling, i.e., the NF-κB [77]. CARD14 is composed of a CARD domain, coiled-coil (C-C) domain, SH3 domain, PDZ domain, and GuK domain [78]. These elements are homologous in CARD14, CARD10, and CARD11, but the distribution of the former is primarily limited to the skin [79].

Upregulation of NF-κB caused by activating mutations in *CARD14* induces the expression of IL-8 and CCL20 [30]. Those chemokines recruit immunocompetent cells, which subsequently promote the Th17 axis and the production of IL-23 by dendritic cells [80].

Heterozygous gain-of-function mutations in *CARD14* have been implicated in the pathogenesis of atypical juvenile PRP (OMIM no. 173200) and GPP with concurrent psoriasis vulgaris (OMIM no. 602723) [5]. An overlap in the clinical features of those entities led to elaborating the joint term of CAPE (locus MIM no. 607211) [50].

##### Adaptor Protein Complex 1 Subunit σ1C Deficiency

*AP1S3* gene encodes adaptor protein complex 1 subunit σ1C [81]. It is a conservative heterotetramer protein participating in intracellular vesicular trafficking. Deficiency of *AP1S3* was shown to cause abnormal TLR3 expression and accumulation of p62 protein leading to disruption of autophagy [82, 82]. As an effect, NF-κB activation and upregulation of IL-36 are seen.

*AP1S3* mutations were detected in patients with generalized pustular psoriasis, palmoplantar pustulosis, and acrodermatitis continua (OMIM no. 616106) [81–83]. Although relatively infrequent, they might coexist with other mutations (e.g., *IL36RN*, *CARD14*) and complicate the genetic background of pustular psoriasis. It seems that *AP1S3* deficiency is the most closely associated with palmoplantar pustulosis [84].

#### Diseases Caused by Other Miscellaneous Mechanisms

##### Mevalonate Pathway Abnormalities

The mevalonate pathway is involved in the biosynthesis of isoprenoids [85]. The latter constitute precursors of various substances involved in cell physiology, e.g., quinones acting as a part of the electron transport chain, sterols forming cell membrane components, and carotenoids [86]. Consequently, the processes of cell growth, division, and differentiation affecting keratinocytes are largely attributed to the mevalonate pathway [87]. Additionally, deficiency of geranyl pyrophosphate constituting a product of the mevalonate pathway possibly leads to inflammasome activation [88]. This mixed influence on the process of keratinization and spontaneous inflammation led to propose porokeratosis associated with the mevalonate pathway abnormalities (*MVK*, OMIM no. 175900; *PMVK*, OMIM no. 175800; *MVD*, OMIM no. 614714; and *FDPS* mutations, OMIM no. 616631) as a member of AiKDs [49].

##### Janus Kinase 1 Hyperactivity

The Janus kinase/signal transducers and activators of transcription (JAK/STAT) pathway is a ubiquitous trait present in all human cells [89]. Triggering the surface receptors leads to JAK phosphorylation and subsequent activation of STATs. The latter modifies the transcription of genes associated with diverse physiological functions, including inflammation. There are 4 isoforms of JAK: JAK1, JAK2, JAK3, and TYK2 [90].

JAK1 is associated with signaling via the interferons, IL-2, IL-6, and IL-10. *JAK1*-activating mutations were detected in hematologic malignancies, such as acute lymphoblastic leukemia [91]. Based on a recent report, heterozygous *JAK1* mutations were also implicated in triggering superficial cutaneous inflammation and aberrant keratosis [92].

##### Proteasome Maturation Protein Deficiency

Proteasome maturation protein (POMP) is encoded by the *POMP* gene [93]. The function of POMP is associated with the maturation of both proteasomes and immunoproteasomes. It is a ubiquitous protein showing expression across all the layers of the epidermis. Decreased assembly of proteasomes associated with POMP abnormalities results in the unfolded protein response and causes endoplasmic stress [94]. Excessive endoplasmic stress can induce aberrant keratinocyte differentiation and apoptosis coexisting with mild superficial lymphohistiocytic infiltrates in histology, thereby fulfilling the criteria of AiKDs. The pathogenic role of endoplasmic stress has been reported in several dermatoses, including psoriasis, reflecting new pathways leading to autoinflammation and the presence of typical cutaneous findings of AiKDs [95].

##### Epidermal Growth Factor Receptor Deficiency

Epidermal growth factor is a molecule orchestrating epidermal maturation and differentiation. It is bound by the epidermal growth factor receptor (EGFR), whose activation decreases the expression of the enzymes responsible for lipid matrix biosynthesis, influences the cornified envelope formation, and downregulates tight junction proteins [96]. EGFR deficiency further causes the upregulation of phospholipase A2, NF-κB, and c-Jun N-terminal kinase 1 stimulating the superficial dermal inflammation [97]. Therefore, it may result in developing a classical AiKD phenotype with concomitant renal and cardiovascular defects reported across the literature [98].

## Clinical Approach to Autoinflammatory Keratinization Diseases

**Fig. 2:**
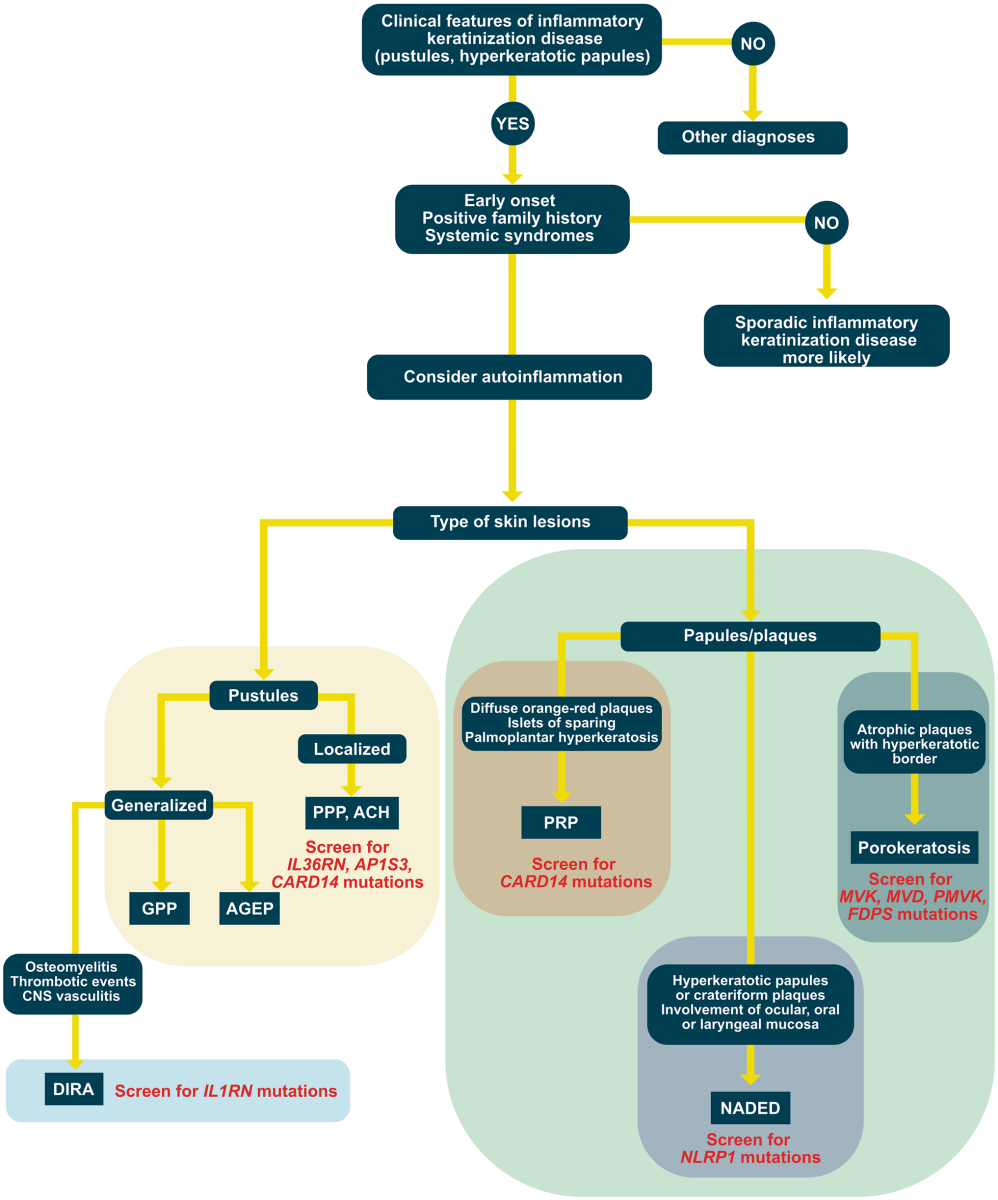
Suggested diagnostic approach to the autoinflammatory keratinization diseases. This figure shows the most significant disorders. ACH—acrodermatitis continua of Hallopeau; AGEP—acute generalized exanthematous pustulosis; DIRA—deficiency of the interleukin-1-receptor antagonist; GPP—generalized pustular psoriasis; NADED—NLRP1-associated autoinflammatory disease with epithelial dyskeratosis; PRP—pityriasis rubra pilaris

As in other autoinflammatory syndromes, establishing the diagnosis of AiKD can be challenging. In most cases of inflammatory keratinization diseases, there is no monogenic mutation resulting in the hyperactivation of innate immunity. At the same time, the clinical features may imitate conventional inflammatory keratinization diseases. Therefore, we propose a clinical approach to the diagnosis and management of AiKDs illustrated in Fig. 2.

First, autoinflammation should be suspected in inflammatory keratinization diseases whenever there is a history of early onset and familial incidence [48]. This should also be considered in the setting of concomitant systemic syndromes such as recurrent fever, arthritis, and cholangitis. However, by definition of AiKDs and in contrast to other autoinflammatory diseases, the inflammation is primarily present in the epidermis and upper dermis [5]. Hence, extracutaneous features involving the nervous system, bones, and gastrointestinal tract are atypical findings. This also explains the difference in the morphology of skin lesions between AiKDs and other autoinflammatory syndromes. Most autoinflammatory diseases characterized by deep cutaneous inflammation can be classified into clinico-pathological patterns such as urticarial dermatosis, neutrophilic dermatosis, or granulomatosis [99]. In contrast, AiKDs present with hyperkeratotic and/or pustular lesions reflecting the superficial inflammation altering the keratinization process.

Once AiKD is considered in the differential diagnoses, the history and physical examination should be detailed to determine specific features of the analyzed case. The morphology, location, and triggers of cutaneous lesions should be carefully studied, along with the possible pattern of inheritance. In most cases, a skin biopsy is recommended to confirm the diagnosis of a suspected inflammatory disorder of keratinization. These established patterns of clinical and histological symptoms should be complemented by genetic studies.

Identification of causative mutations can tailor the treatment regimen to the particular case. In contrast to classic systemic treatments such as methotrexate and oral retinoids, most AiKDs will respond to pharmaceuticals targeting the IL-1, IL-12/IL-23, or IL-17 pathways [48, 50]. For example, favorable effects of treatment with IL-1 receptor antagonist anakinra were reported in a patient with generalized pustular psoriasis associated with *IL36RN* mutation [100]. The IL-1R blockade is not commonly used in classic forms of inflammatory keratinization diseases. Hence, effort should be made to establish the possibility of expanding the available therapeutic options and improve the management of patients with AiKDs.

Below we provide a brief, clinically oriented description of the most significant AiKDs (Tables 1 and 2).

**Table 1:** Autoinflammatory keratinization diseases described in the present article

Disease name	Clinical findings	Primary affected gene	Mode of inheritance	OMIM no.
Generalized pustular psoriasis without concomitant psoriasis vulgaris	• Recurrent crops of sterile pustules occupying large body surface area (tendency for erythroderma) • Accompanied by systemic symptoms (fever, malaise, uveitis, osteoarthritis, cholangitis) and elevated biomarkers of systemic inflammation • Confluence of pustular lesions may result in epidermal detachment causing electrolyte abnormalities and increased risk of infection	*IL36RN*	Autosomal recessive	605507
Impetigo herpetiformis	• Generalized pustular psoriasis in pregnant women (onset usually in the 3rd trimester) • Possible fetal distress resulting from placental insufficiency	*IL36RN*	Autosomal recessive	605507
Generalized pustular psoriasis with concomitant psoriasis vulgaris	• Clinical picture involves widespread pustular lesions which may coexist with typical lesions of psoriasis vulgaris • The clinical course is frequently more benign than in IL-36Ra deficiency	*CARD14*	Autosomal recessive	602723
Palmoplantar pustulosis	• Pustular lesions superimposed on erythematous background localized on the palms and/or the soles • May coexist with onychodystrophy and psoriasis vulgaris • Chronic course	*AP1S3*	Autosomal recessive	616106
Acrodermatitis continua Hallopeau	• Pustular lesions localized on the distal digits of the hands and/or the feet • Frequent nail involvement • Severe cases may be associated with osteitis of the distal phalanges	*IL36RN*	Autosomal recessive	616106
Deficiency of interleukin-1 receptor antagonist (DIRA)	• Generalized pustular lesions • May be associated with systemic involvement (aseptic osteomyelitis, thrombotic events, and central nervous system vasculitis) • Possible pathergy • Marked elevation in acute phase proteins	*IL1RN*	Autosomal recessive	612852
Type V pityriasis rubra pilaris	• Hyperkeratotic papules coalescing into diffuse orange-red plaques with characteristic ‘islets of sparing’ • Typically coexists with palmoplantar keratoderma • Early age of onset, chronic course, resistance to treatment	*CARD14*	Autosomal dominant	https://www.omim.org/entry/173200
CARD14-associated papulosquamous eruption (CAPE)	• Overlap of CARD14-associated pityriasis rubra pilaris and psoriasis vulgaris • Prominent involvement of the cheeks, chin, and ears, early age of onset, positive family history of psoriasis or pityriasis rubra pilaris, minimal response to conventional topical and systemic psoriasis therapies, and improvement with targeted treatments (ustekinumab)	*CARD14*	Autosomal dominant	locus MIM697211
Porokeratosis	• Single or multiple atrophic plaques surrounded by a hyperkeratotic ridge-like border	*MVK, MVD, PMVK, FDPS*	Autosomal dominant disorder with somatic lesional second hit mutation	175900*;* 175800*;* 614714; 616631
JAK1-associated autoinflammatory keratinization disease with hepatitis and autism	• Widespread ichthyotic eczema • Extracutaneous manifestations: rapidly progressing hepatic cirrhosis, growth retardation, motor impairment, learning disability and hyperlipidemia • Only 1 case described to date, supported by similar cutaneous symptoms in JAK1 hyperactivation models	*JAK1*	Autosomal dominant	618999
Keratosis linearis with ichthyosis congenita and sclerosing keratoderma (KLICK)	• Palmoplantar keratoderma, hyperkeratotic plaques (often in a linear distribution), ichthyosiform scaling, circular constrictions around the fingers, and multiple linear papular lesions in the arm folds and on the wrists	*POMP*	Autosomal recessive	601952
Epidermal growth factor receptor deficiency	• Papulo-pustular rash, loss of scalp hair, trichomegaly • Possible systemic involvement (gastrointestinal and respiratory symptoms (watery diarrhea, respiratory difficulties, and bronchiolitis))	*EGFR*	Autosomal recessive	131550

The spectrum of NLRP1-associated autoinflammatory disease with epithelial dyskeratosis is summarized in a separate table

**Table 2:** Summary of diseases associated with NLRP-1 hyperactivation. Common pathogenesis and clinical similarities justify considering these entities jointly within the spectrum of NLRP1-associated autoinflammatory disease with epithelial dyskeratosis (NADED)

Disease name	Clinical findings	Histology	Mode of inheritance
Familial keratosis lichenoides chronica	• Multiple keratotic papules in a pattern distribution (linear, reticulate) coalescing into plaques, seborrheic dermatitis-like lesions on the face, palmoplantar hyperkeratosis, hypertrophic nail deformation • Associated with arthritis	• Patchy interface dermatitis with vacuolar degeneration and numerous necrotic keratinocytes along the dermoepidermal junction, foci of acanthosis, wedge-shaped hypergranulosis, and keratotic plugging of the infundibulum	Autosomal codominant
NLRP1-associated autoinflammation with arthritis and dyskeratosis	• Dyskeratotic cutaneous lesions with phrynoderma, filiform hyperkeratosis, palmoplantar symmetrical hyperkeratosis, and papules with pseudocomedones • Associated with recurrent fever, arthritis, elevated markers of inflammation, vitamin A deficiency, and dental dysplasia in some patients	• Acanthosis, variable amount of dyskeratotic keratinocytes, dense orthokeratosis with foci of parakeratosis; dyskeratosis may almost completely replace the granular layer	Autosomal dominant/ autosomal recessive
Multiple self-healing palmoplantar carcinoma	• Rapidly growing, ulcerative-nodular palmoplantar tumors; most heal spontaneously within 6 weeks leaving pitted scars • Associated with nodular conjunctival lesions suggestive of keratoacanthoma, thickened nails, hyperkeratosis pilaris, and increased risk of epithelial tumors	• Circumscribed acanthosis, hyperkeratosis, dyskeratosis and papillomatosis, variable lichenoid infiltrate, and pigment incontinence • Possible progression into invasive SCC	Autosomal dominant
Juvenile recurrent respiratory papillomatosis	• Laryngeal papillomas • Mild cutaneous involvement: *Atrophoderma vermiculata* on the cheeks, plantar warts, and keratosis pilaris	• Cutaneous lesions were not examined • Laryngeal papillomas show acanthosis, moderate hyperkeratosis, areas of binucleated cells, and focal presence of koilocytes associated with primarily lymphocytic inflammatory infiltrate in the lamina propria	Autosomal recessive
Corneal intraepithelial dyskeratosis	• Primarily ocular and laryngeal manifestations (conjunctival and corneal plaques possibly causing vision impairment, laryngeal papillomas) • Palmoplantar hyperkeratosis, pruritic hyperkeratotic scars, dyshidrosis, nail dystrophy	• Cutaneous lesions were not examined • Corneal and laryngeal lesions show epithelial hyperplasia, acanthosis, parakeratosis, and suprabasal dyskeratosis	Autosomal dominant

### Generalized and Localized Variants of Pustular Psoriasis

#### Clinical Features

**Fig. 3:**
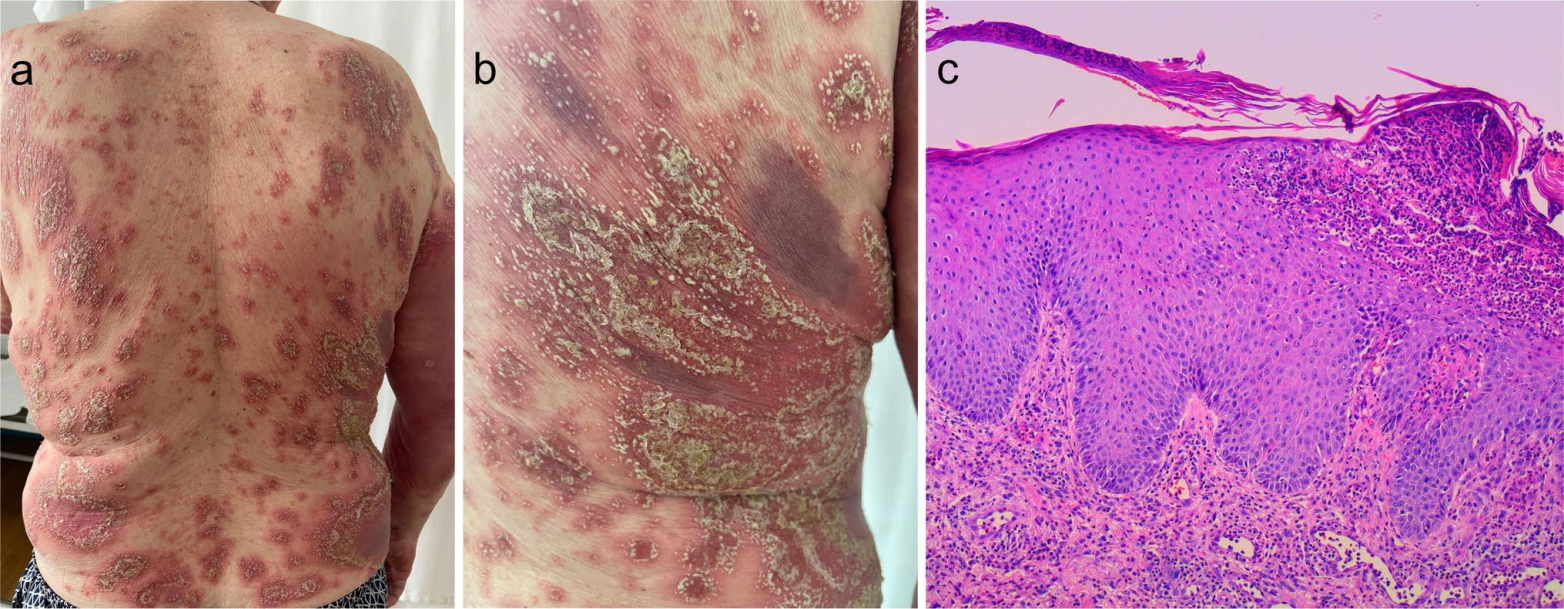
Sterile pustules in generalized pustular psoriasis. **a**, **b** Histology shows Munro microabscesses and spongiform pustules of Kogoj **c** occasionally accompanied by typical features of psoriasis vulgaris (acanthosis, confluent parakeratosis, elongation of rete ridges, and dilation of papillary blood vessels)

**Fig. 4:**
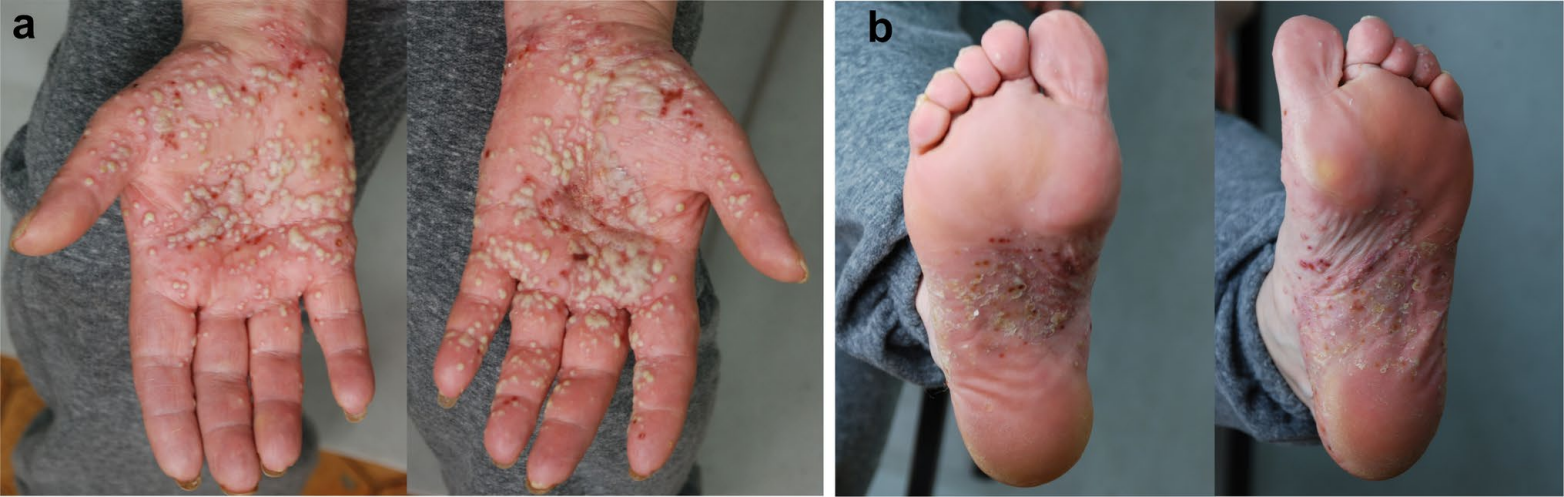
Palmoplantar pustulosis. Sterile pustules limited to palms (**a**) and/or soles (**b**) which may rupture and produce fissures and erosions, thereby impairing fine motor skills and walking

**Fig. 5:**
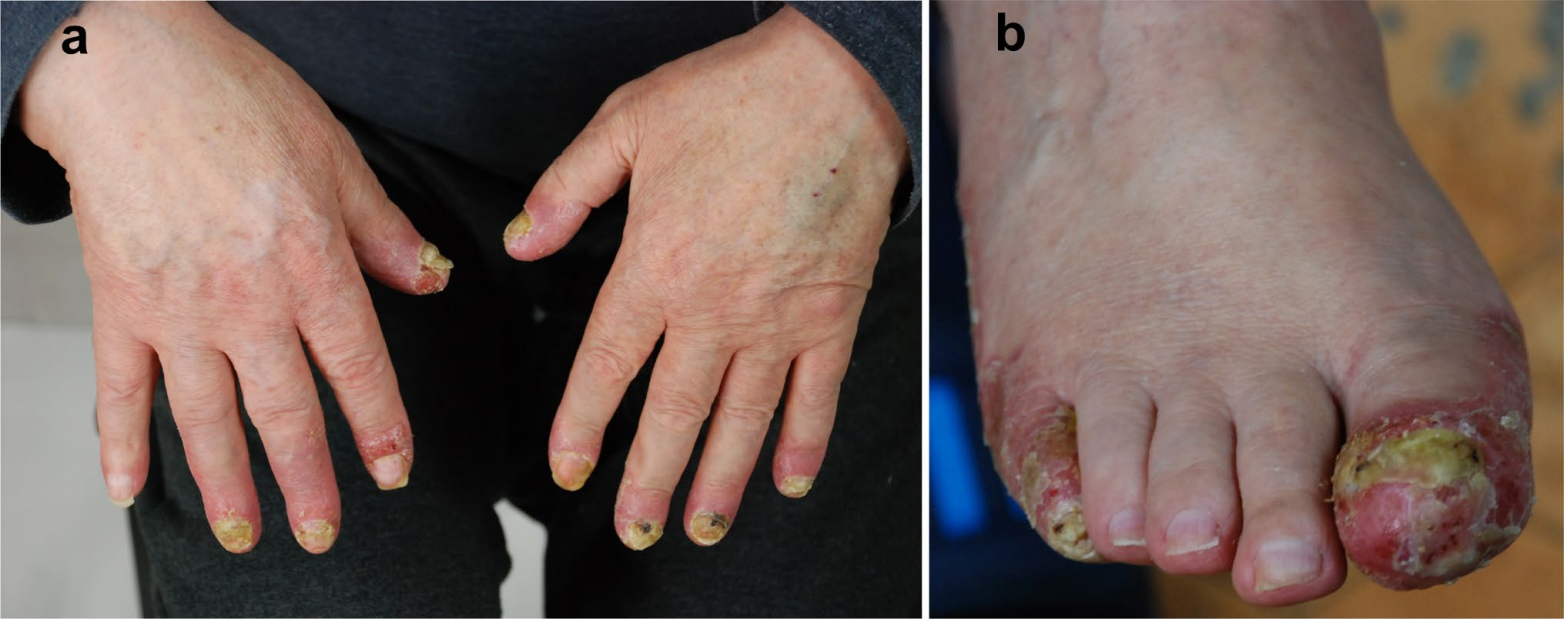
Acrodermatitis continua of Hallopeau. Pustular lesions occupying the distal phalanges of the hands (**a**) and/or the feet (**b**). There is a prominent involvement of the nail apparatus resulting in onychodystrophy

Psoriasis is a frequent inflammatory keratinization disease characterized by a chronic course and a significant negative effect on the quality of life [47]. It is estimated that the prevalence of psoriasis amounts to 3% of the general population [47, 101]. Psoriasis can be divided into two primary subtypes: the more frequent nonpustular type (approx. 90% of cases) presenting with papulosquamous lesions and the less prevalent pustular type. The spectrum of pustular psoriasis involves generalized forms (the acute von Zumbusch variant (Fig. 3), impetigo herpetiformis, annular pustular psoriasis, and juvenile pustular psoriasis) and localized forms (palmoplantar pustulosis (Fig. 4) and acrodermatitis continua of Hallopeau (Fig. 5). Most pustular psoriasis subtypes can present as AiKD [5].

The diagnosis of psoriasis is clinical [101]. In doubtful cases, histopathological examination can be performed to confirm the clinical suspicion. According to the name, the pustular forms of psoriasis are characterized by the formation of sterile pustules. The von Zumbusch variant is typically accompanied by systemic symptoms (fever, malaise, uveitis, osteoarthritis, cholangitis) and elevated biomarkers of systemic inflammation. It is also a potentially lethal condition, threatening with quick progression of cutaneous lesions into erythroderma. The confluence of pustular lesions may result in epidermal detachment causing electrolyte abnormalities, increased risk of infection, and other serious complications [102]. The localized variants are rarely associated with systemic manifestations but tend to present a protracted course [101].

Impetigo herpetiformis is considered to be a form of GPP developing during pregnancy [65, 102]. In most cases, the lesions begin during the third trimester. Similarly to GPP, impetigo herpetiformis is a potentially life-threatening condition. Apart from the risk to the mother, it is associated with possible fetal distress resulting from placental insufficiency [103]. Hence, the prompt diagnosis and treatment are essential to prevent severe maternal and fetal complications.

Palmoplantar pustulosis constitutes a localized variant of psoriasis affecting the palms and/or the soles [104]. The clinical picture involves persistent eruptions of sterile pustules superimposed on an erythematous and desquamative background. The prevalence of PPP is estimated at up to 0.05% of the general population and is slightly higher in women [105]. The lesions may coexist with typical lesions of psoriasis vulgaris in other locations and nail changes (onycholysis, pitting, nail destruction) [106].

Acrodermatitis continua of Hallopeau (ACH) is a very rare form of localized pustular psoriasis distinguished by the presence of lesions on the distal digits of the hands and/or the feet and involvement of the nail apparatus resulting in onychodystrophy (Fig. 5) [102, 107]. It may also be associated with osteitis of the distal phalanges. ACH has been reported in patients suffering from occasional GPP flare-ups, which supports the view that those two diseases belong to the common spectrum [102].

Acute generalized exanthematous pustulosis (AGEP) is the main differential diagnosis of GPP [108]. AGEP is a widespread pustular drug reaction driven by drug-specific CD4 and CD8 lymphocytes [109]. The skin lesions have an almost identical morphology and progression as in GPP. Small pustules superimposed on an erythematous base are initially seen in the skin folds and may quickly spread to other skin areas. AGEP can be associated with systemic symptoms (fever, malaise) and laboratory abnormalities including elevated C-reactive protein, hypocalcemia, hypoalbuminemia, and leukocytosis (with concomitant eosinophilia in approximately 30% of cases) [109]. The offending drugs are usually administered 48 h prior to the onset of symptoms. Identification of the common genetic background of GPP and AGEP raised controversies about whether AGEP is not indeed a form of drug-induced GPP [66].

Another differential diagnosis of GPP is the subcorneal pustular dermatosis (SPD), also known as the Sneddon-Wilkinson syndrome [110]. SPD is characterized by the presence of flaccid hypopyon pustules with annular distribution. Similarly to GPP and AGEP, the lesions tend to arise in the intertriginous areas and may progress to involve the trunk and extremities. SPD tends to run a relatively benign and self-limiting course. Systemic symptoms are infrequent. It usually begins in adulthood, but several cases of childhood-onset cases have also been reported [111, 112]. Clinically, SPD may imitate IgA pemphigus, but it is differentiated by negative direct and indirect immunofluorescence studies [113]. It was shown that SPD may coexist with a number of other disorders, involving connective tissue diseases (rheumatoid arthritis, systemic lupus erythematosus) and hematologic disorders (IgA monoclonal gammopathy, multiple myeloma) [110]. To date, the pathogenesis of SPD is unknown.

#### Histology

The histological features of pustular psoriasis involve the presence of Munro microabscesses and spongiform pustules of Kogoj (Fig. 3c) [101]. The lesions can coexist with features of psoriasis vulgaris (acanthosis, confluent parakeratosis, elongation of rete ridges, and dilation of papillary blood vessels) or be present in the setting of otherwise unchanged epidermis.

#### Genetic Background

**Fig. 6:**
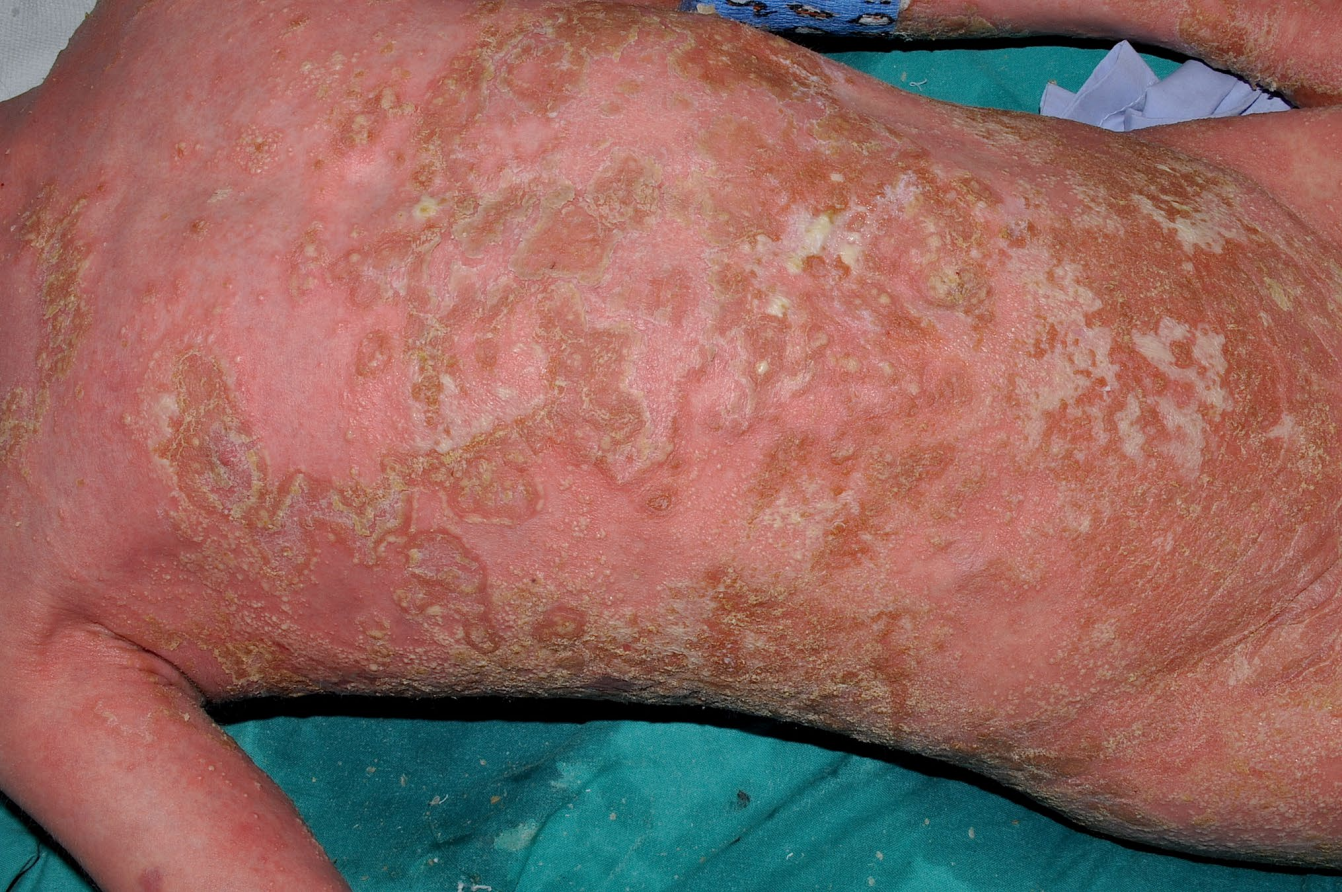
Generalized pustular lesions in a child with confirmed deficiency of the IL-36 receptor antagonist (DITRA)

As discussed previously, autoinflammatory variants of generalized and localized pustular psoriasis can result from several pathogenetic pathways, i.e., *IL36RN* loss-of-function (OMIM no. 605507), *CARD14*-activating (OMIM no. 602723), and *AP1S3* loss-of-function (OMIM no. 616106) pathogenic variants. The IL-36Ra deficiency results from biallelic loss of function variants in *IL36RN* [61]. The patients with IL-36Ra deficiency present a more severe phenotype and have an earlier onset of GPP compared to those with *CARD14* variants (Fig. 6) [108]. *CARD14-*activating variants stimulate the transcription of pro-inflammatory factors in the NF-κB-dependent pathway. This can lead to the onset of pustular psoriasis in patients with concurrent psoriasis vulgaris. Other clinical features involve early age of onset; prominent involvement of the cheeks, chin, and ears; family history of psoriasis or PRP; and minimal improvement using classic therapies of psoriasis [50]. Lastly, *AP1S3* variants seem most closely associated with palmoplantar pustulosis [84].

Recently, the role of *IL36RN* variants was shown to underlie most cases of impetigo herpetiformis particularly in the East Asian populations [114]. This supports the hypothesis on a common pathogenetic background of impetigo herpetiformis and GPP. *IL36RN* variants were also detected in patients with AGEP, substantiating the already difficult differentiation with GPP [118].

#### Treatment

GPP is an infrequent entity. Therefore, large randomized controlled trials assessing the efficacy of different treatment modalities are lacking [101, 119]. There is even less data regarding autoinflammatory cases of GPP caused by *IL36RN* and *CARD14* mutations.

Generally, it is recommended to treat acute severe GPP with cyclosporine or infliximab [120]. In more chronic cases, systemic retinoids (acitretin) and methotrexate can be considered. However, patients with autoinflammatory forms of pustular psoriasis may be recalcitrant to standard treatment modalities [50]. Furthermore, TNF inhibition with infliximab was shown to induce paradoxical palmoplantar pustulosis in a subset of patients with psoriasis vulgaris [121]. Therefore, off-label use of other biologics should be considered.

Based on experimental data, patients with *IL36RN* variants could benefit from the blockade of IL-36R signaling. This assumption was recently tested in a proof-of-concept study of spesolimab, an anti-IL-36R antibody which showed high efficacy after single-dose administration [122]. Spesolimab has been recently approved by the US Food and Drug Administration and the European Medicines Agency for the treatment of GPP flares in adults [123, 124]. Imsidolimab is another biologic targeting IL-36R under investigation in GPP [125]. In a clinical trial of palmoplantar pustulosis, imsidolimab failed to meet the primary outcomes.

Considering that both IL-36Ra deficiency and CARD14 hyperactivation induce inflammatory cascade involving Th1 and Th17 molecules, inhibition of those signals could be associated with favorable clinical outcomes. Indeed, patients with those variants were shown to improve after administration of biologics targeting IL-12/IL-23 (ustekinumab), IL-17 (secukinumab), and TNF (etanercept, infliximab, adalimumab) [119].

With respect to impetigo herpetiformis, most recent reports of cases refractory to conventional treatment (i.e., topical steroids, cyclosporine, and phototherapy) showed a favorable response to secukinumab [115–117]. TNF inhibitors, particularly certolizumab for its safety in pregnancy, also seem to be a potentially beneficial treatment strategy [102].

It is important to mention that systemic steroids can induce GPP exacerbations, especially if rapidly tapered. However, some data support their cautious use as adjuvant treatment in cases associated with systemic symptoms or arthritis. For example, the Japanese guidelines for the management of GPP advocate oral steroids particularly in pregnancy, during which many drugs are contraindicated because of their teratogenic potential [126].

### Deficiency of Interleukin-1 Receptor Antagonist (DIRA)

#### Clinical Features

DIRA is a rare, severe disorder associated with uncontrolled signaling via the IL-1R due to the deficiency of its negative regulator, the IL-1Ra [67, 68, 127].

DIRA usually starts in the neonatal period and has a dramatic course resulting in a mortality of up to 30% in untreated infants [127]. The clinical features involve a pustular rash imitating GPP, aseptic multifocal osteomyelitis, thrombotic events, and central nervous system vasculitis [67, 127]. In longstanding, non-treated disease hyperostosis may be seen. Additional disease stigmata involve elevation in acute phase proteins and pathergy.

#### Histology

The findings on the cutaneous biopsy are identical to GPP. However, the inflammatory infiltrates may contain even larger amounts of neutrophils [67, 128].

#### Genetic Background

DIRA (OMIM no. 612852) is caused by biallelic loss-of-function pathogenic variants in *IL1RN* [127]. To date, 10 different variants have been reported. In the largest case series described to date, Aksentijevich et al. [129] presented 1 patient from Newfoundland who was homozygous for a deletion of 2 bp (c.156_157delCA) causing a frameshift mutation, three patients from families of Dutch ancestry homozygous for a nonsense mutation affecting the amino acid at position 77 (nucleotide mutation, c.229G → T; resultant amino acid mutation, E77X), two patients from a consanguineous Lebanese family homozygous for a nonsense mutation (c.160C → T), and one patient from Puerto Rico homozygous for a deletion of approximately 175 kb on chromosome 2q affecting six interleukin-1-related genes. Reportedly, the symptoms varied in terms of pustular rash severity and internal organ involvement even in patients with the same genotypes, which underscores the probable effect of environmental factors on the genotype-phenotype correlation.

#### Treatment

The successful treatment of DIRA can be achieved by blocking the IL-1 inflammatory pathway. Among the possible treatment options are anakinra (recombinant IL-1Ra), canakinumab (an anti-IL-1β monoclonal antibody), and rilonacept (a soluble decoy IL-1R) [68, 130–132]. Treatment with those agents was shown to be effective in improving the balance between pro- and anti-inflammatory signaling via IL-1R and constricting the autoinflammatory cascade in DIRA.

### Nucleotide-Binding Oligomerization Domain-Like Receptor Containing a PYRIN Domain 1 (NLRP1)-Associated Autoinflammatory Disease With Epithelial Dyskeratosis (NADED)

As discussed above, NLRP1 inflammasomes are key structures orchestrating the immune response in the epithelia [133]. Akiyama et al. distinguished familial keratosis lichenoides chronica (KLC) as the prototypic AiKD associated with activating mutations in *NLRP1* [5]. However, more cases of NLRP1 hyperactivation characterized primarily by skin and mucosal inflammation and variable systemic involvement have been described (Table 2). Similarities in terms of clinical presentation, histology, and pathogenesis justify expanding this group and distinguishing it as a separate endotype of AiKDs. Therefore, we propose a joint term of NLRP1-associated autoinflammatory disease with epithelial dyskeratosis (NADED) which encompasses the whole spectrum of AiKDs secondary to NLRP1 hyperactivation.

#### Clinical Features

The onset of NADED is usually during infancy or early childhood [18, 74–76, 133–135]. The patients may present cutaneous, oral, laryngeal, and/or ocular symptoms.

**Fig. 7:**
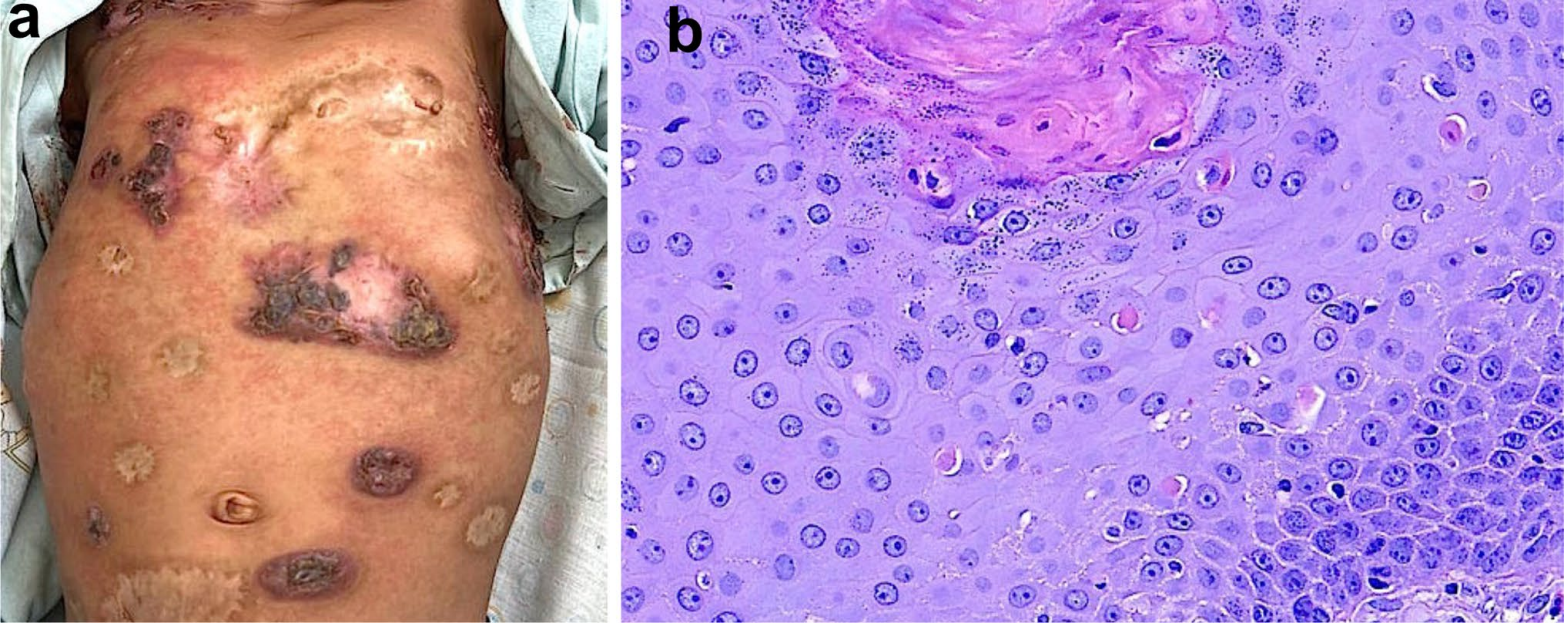
NLRP1-associated autoinflammatory disease with epithelial dyskeratosis (NADED). The patient presented dyskeratotic cutaneous lesions in the form of crateriform plaques, which resolved with scarring (**a**). Histology showed numerous dyskeratotic cells and acanthosis (**b**). Photos by courtesy of Dr. Felipe Velásquez

Cutaneous lesions vary in size from small papules to large crateriform plaques resembling keratoacanthomas [133, 135, 136]. The lesions are hyperkeratotic and may partially resolve with scarring (Fig. 7a). Recurrent erythematous suppurative papules and plaques have also been described. The Koebner phenomenon, e.g., following cutaneous biopsies, has been reported [134]. The lesions favor the trunk, buttocks, and extremities and usually spare the face and head area. They may be arranged in a pattern distribution (linear, reticulate) [137]. The patients may complain of itching or pain.

Palmoplantar punctate or papular hyperkeratotic lesions are a typical finding [74–76, 135]. In several pedigrees, recurrent, spontaneous formation of well-differentiated squamous cell carcinomas with a surprising tendency for self-resolution was reported [136]. Hypertrophic nail deformation, dyshidrosis, atrophoderma vermiculata, and severe atopy may also be seen with variable frequency [136, 138–142]. Based on available data, it seems that FKLC is indeed a mild form of NADED.

Apart from cutaneous lesions, the patients may also develop mucosal involvement [18, 75, 76, 133, 134]. Ocular symptoms are reported most frequently and include photophobia, conjunctivitis, corneal dyskeratosis, keratopathy with neovascularization, and, in the most severe cases, complete corneal opacification [18, 75, 76, 135, 140]. Oral involvement can manifest with leukoplakia, swelling of the gingival mucosa, dental dysplasia, and alveolysis [75, 134, 135, 143, 144]. The latter may lead to early tooth loss. Furthermore, patients may present multiple laryngeal papillomas and dysphonia [74, 142, 145].

NADED symptoms are usually limited to the epithelia. However, some patients, particularly those with mutations within the FIIND and linker domains, present systemic symptoms [133, 134]. These involve recurrent fever, oligoarticular arthritis, uveitis, growth retardation, hepatosplenomegaly, autoimmune hemolytic anemia, and thyroiditis. Laboratory tests may reveal elevated CRP, low vitamin A, and anti-nuclear and antiparietal antibodies.

#### Histology

Reported histological features of the cutaneous and mucosal lesions in NADED were quite uniform across different studies [76, 133, 134]. Smaller papular lesions tend to show unspecific findings such as irregular acanthosis, orthohyperkeratosis interrupted by focal parakeratosis, hypergranulosis, and variable lichenoid infiltrate along the dermo-epidermal junction. Larger plaque and crateriform lesions show massive orthohyperkeratosis with focal parakeratosis, acanthosis, and numerous dyskeratotic cells with a tendency to replace the granular layer in some specimens (Fig. 7b). In cases with oncogenic transformation, typical features of well-differentiated squamous cell carcinoma may be seen [136]. Mucosal lesions show comparable features, i.e., acanthosis, focal parakeratosis, dyskeratosis, and primarily lymphocytic inflammatory infiltrate in the corium and lamina propria [74, 135].

#### Genetic Background

To date, more than 30 cases of NADED have been described worldwide [74–76, 133–135]. Reported modes of inheritance involve autosomal recessive, autosomal dominant, or autosomal codominant (Table 2). The underlying mutations have been mapped to PYD, FIIND, LRR, and linker domains of NLRP1, most likely resulting in the disruption of the auto-inhibitory domain of NLRP1 [18]. A prominent intrafamilial clinical heterogeneity reported in NADED suggests the effect of largely unknown genetic, epigenetic, and environmental modifying factors. This was partly elucidated by Li et al. [76] who described two siblings sharing the same *NLRP1* gene variant resulting in a p.Leu813Pro substitution of the LRR domain who presented significantly different phenotypes. The younger sister had generalized inflammatory nodules with keratotic plugs reminiscent of multiple keratoacanthomas, while the older sister showed lesions compliant with familial keratosis lichenoides chronica. The authors attributed those differences to additional genomic variants associated with atopy and psoriasis and denoted IL-5 and IL-17 as the most probable cofactors of severe cutaneous inflammation in the younger sister.

#### Treatment

Currently, there are no targeted treatment options for NADED. Steroids, retinoids, and TNF inhibitors were generally reported as unsuccessful [133, 135]. Treatments targeting IL-1 were used in four cases yielding mixed results. Systemic anakinra, later switched to canakinumab, considerably improved systemic symptoms in one patient [133]. In another study, a combination of systemic canakinumab and eye drops with anakinra showed favorable outcomes with respect to oral and ocular involvement [75]. However, another study reported a lack of effect of canakinumab in two siblings with NADED [76].

### Pityriasis rubra pilaris

#### Clinical Features

**Fig. 8:**
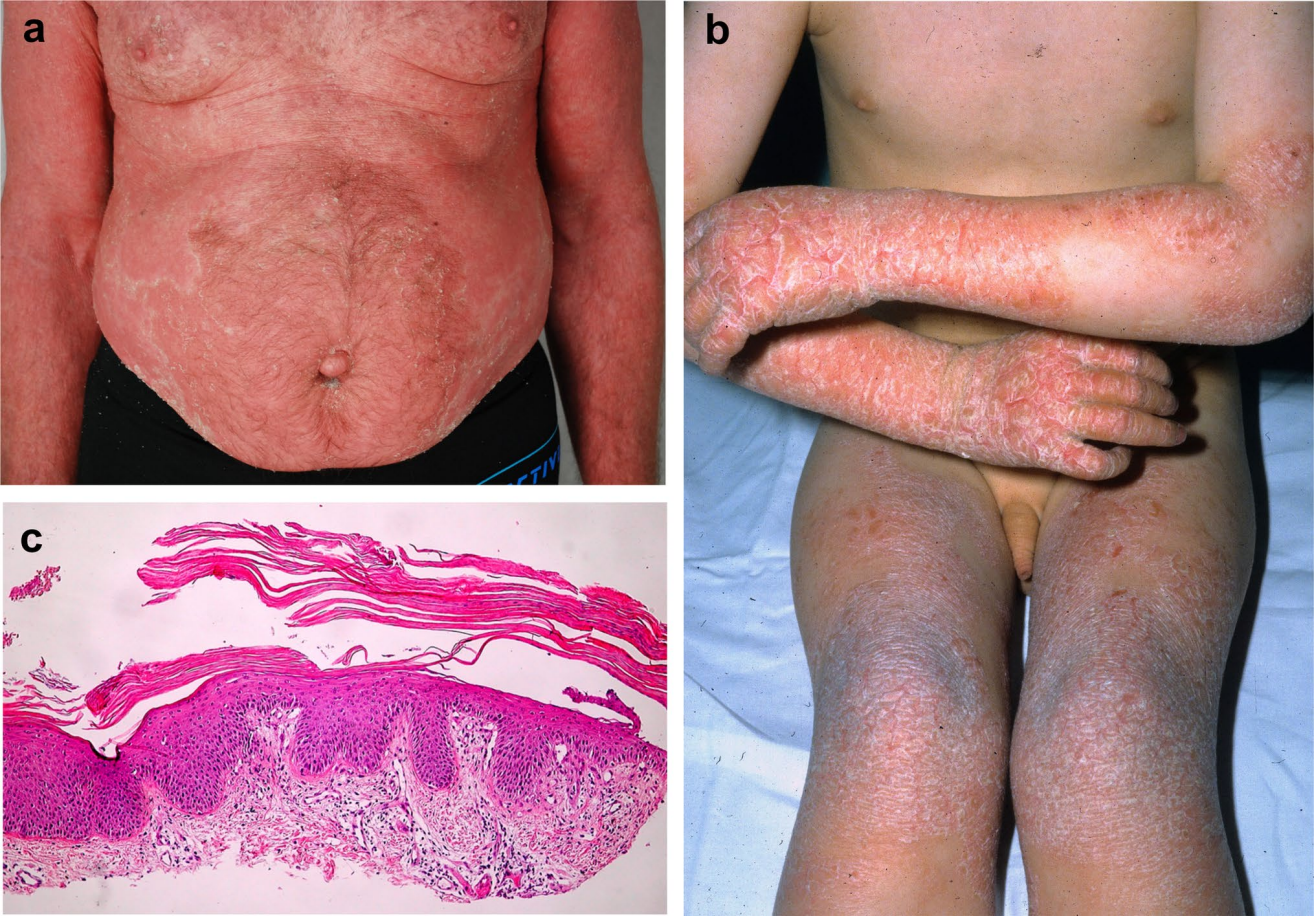
Pityriasis rubra pilaris and CARD14-associated papulosquamous eruption (CAPE). Patient with pityriasis rubra pilaris presented typical diffuse hyperkeratotic papules which progressed to erythroderma with characteristic islets of sparing, i.e., foci of uninvolved skin (**a**). The second patient with CARD14-activating mutation showed lesions mimicking psoriasis (**b**). Histology of pityriasis rubra pilaris revealing follicular plugging, preserved granular layer or hypergranulosis, and alternating areas of ortho- and parakeratosis (**c**)

Pityriasis rubra pilaris is a papulosquamous inflammatory dermatosis sharing certain pathogenetic and clinical similarities with psoriasis [32, 33]. PRP is an infrequent entity, but its exact incidence is uncertain. It is estimated that PRP is diagnosed in approximately 1 in 5000–50,000 adult patients and 1 in 500 pediatric patients presenting with skin disease [146]. PRP is characterized by hyperkeratotic papules, which tend to coalesce into diffuse orange-red plaques (Fig. 8a). A hallmark of PRP involves the presence of small areas of non-involved skin referred to as “islets of sparing” in between those plaques [147]. Another characteristic feature is the presence of palmoplantar keratoderma.

In 1980, Griffiths proposed a classification of PRP into five subtypes adopting the morphology of skin lesions, age of onset, and prognosis as the primary criteria [146]. Among those subtypes, type V PRP represents the atypical juvenile type comprising most cases of familial PRP and around 5% of total PRP cases. Type V PRP is characterized by an early onset and chronic, recalcitrant course. Follicular hyperkeratosis and ichthyosiform dermatitis are common findings, while some patients also exhibit sclerodermatous changes in their hands and feet.

#### Histology

Histology of PRP typically demonstrates psoriasiform hyperplasia, follicular plugging, lack of epidermal microabscesses, and intact granular layer or hypergranulosis, with the latter three features differentiating it from psoriasis (Fig. 8c) [147, 148]. Additionally, instead of confluent parakeratosis, PRP shows a vertical and horizontal pattern of orthokeratosis alternating with spotty parakeratosis. In the papillary dermis, vascular ectasia is usually seen, albeit not as prominently as in psoriasis. Mild dermal perivascular lymphohistiocytic infiltrate can be present.

#### Genetic Background

Most cases of PRP are not associated with a monogenic autoinflammatory predetermination. Familial PRP is attributed to gain-of-function variants in *CARD14* which results in NF-κB activation and transcription of pro-inflammatory cytokines, primarily IL-17, IL-22, and IL-23 [78]. Analysis of inheritance in the initial report revealed an incomplete penetrance in some of the analyzed families, which implies that the phenotypic expression of these causative variants could be modified epigenetically or by environmental factors. In the skin, NF-κB promotes keratinocyte viability during differentiation [149]. In line with that, mice with hyperactivation of NF-κB develop generalized papulosquamous skin lesions.

#### Treatment

Familial PRP and psoriasis due to *CARD14* variants form one spectrum known as *CARD14*-associated papulosquamous eruption (CAPE) (Fig. 8b) [50]. The shared molecular background ensues analogic treatment strategies. Classic treatments such as acitretin and methotrexate may be associated with lower success rates than in sporadic PRP [150].

Transcriptional responses of NF-κB are induced primarily by TNF. However, this pathway seems less suitable for a therapeutic target in case of sporadic NF-κB activation due to *CARD14* mutations [150, 151]. Indeed, TNF inhibitors showed unsatisfactory therapeutic effects in CAPE. Most credible data on targeted treatments point to the beneficial effect of ustekinumab [50]. Case reports further suggest a possible role of IL-17 inhibition with secukinumab and ixekizumab [152, 153].

### Porokeratosis

#### Clinical Features

Porokeratosis refers to a heterogenous group of keratinization diseases manifesting by single or multiple atrophic plaques surrounded by a hyperkeratotic ridge-like border [87, 154]. It is an infrequent entity characterized by a slight male predominance. The lesions of porokeratosis favor the extremities. Importantly, long-standing porokeratosis is a risk factor for the development of cutaneous cancers.

**Fig. 9:**
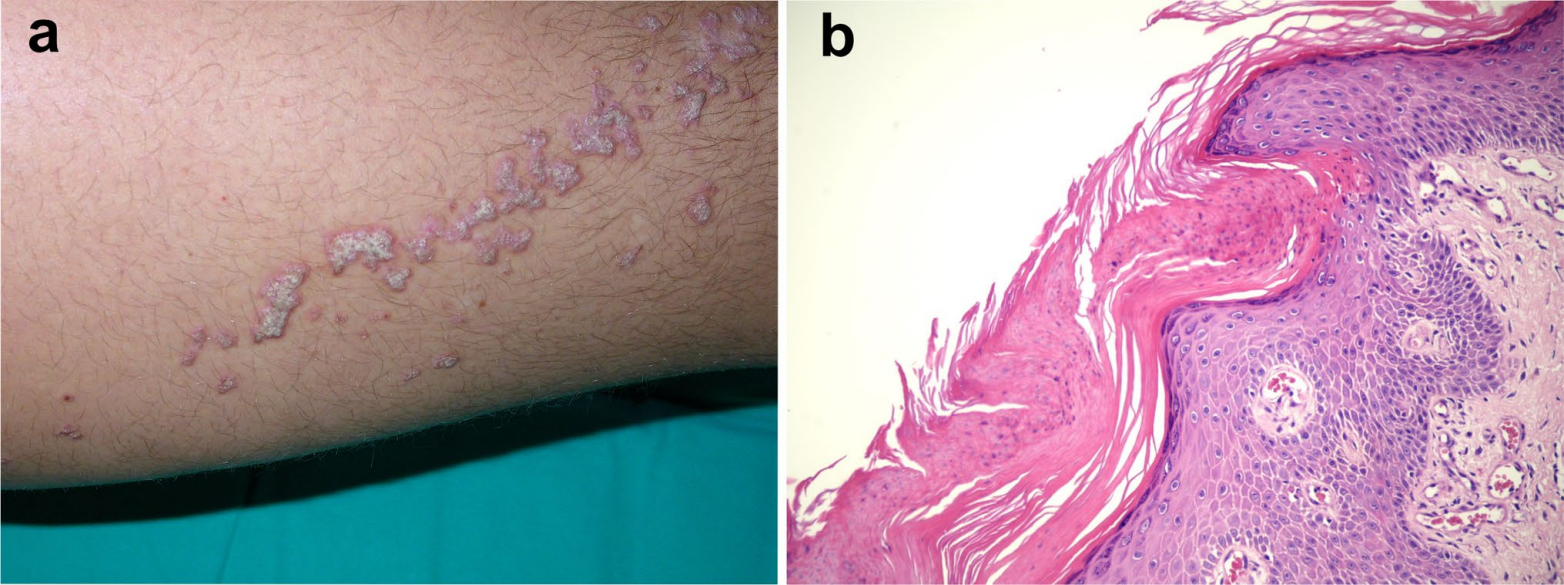
Linear porokeratosis—segmental hyperkeratotic plaques in a child (**a**). In histology, the most typical phenomenon is the presence of cornoid lamellae, i.e., thin columns of parakeratotic cells overlying foci of hypogranulosis and vacuolated keratinocytes (**b**)

The common clinical variants are grouped into localized (classical porokeratosis of Mibelli, linear porokeratosis, punctate porokeratosis, solar facial porokeratosis, and genital porokeratosis) and generalized (disseminated superficial porokeratosis, disseminated superficial actinic porokeratosis, and disseminated palmoplantar porokeratosis) [154]. Currently, the crucial differentiation between spontaneous and AiKD porokeratosis cases is based on the presence or absence of the segmental or mosaic superimposed distribution (Fig. 9a) [155–157]. In their paper proposing porokeratosis as an AiKD, Takeichi and Akiyama exemplified the rare eruptive pruritic papular porokeratosis [87]. It is characterized by suddenly appearing itchy lesions that resolve spontaneously within months with post-inflammatory hyperpigmentation. Description of all the variants exceeds the scope of this review.

#### Histology

The most typical histologic feature of porokeratosis is the presence of cornoid lamellae (Fig. 9b) [49, 154]. These structures can be described as tightly packed thin columns of parakeratotic cells. Cornoid lamellae overlie foci of hypogranulosis and vacuolated keratinocytes. These features are accompanied by the occasional presence of dyskeratotic keratinocytes below the spinous layer and superficial dermal inflammation of variable density.

#### Genetic Background

A recent study analyzing seven families with porokeratosis revealed novel causative genetic variants in the mevalonate pathway [158]. The genes *MVK*, *MVD*, *PMVK*, and *FDPS* encode mevalonate kinase, mevalonate decarboxylase, phosphomevalonate kinase, and farnesyl diphosphate synthase, respectively [154, 159]. They are involved in the synthesis of isoprenoids constituting essential intermediate products of cholesterol and sterol biosynthesis. Downregulation of isoprenoids affects cell growth and differentiation. Porokeratosis (OMIM nos. 175900; 175800; 614714; 616631) is an autosomal dominant disorder developing as a result of second-hit somatic mutation causing loss of heterozygosity [160]. This process occurs in carriers of germline dominant mutation inherited from one of the parents when another acquired mutation affecting wild-type allele appears solely in the keratinocytes [159]. The latter is mostly attributed to UVA-induced mutagenesis, which explains the photodistribution of lesions in most cases of porokeratosis. As *MVK* is expressed not only in keratinocytes, but also leukocytes, loss-of-function mutations in both alleles of this gene can result in an onset of systemic diseases, such as the hyper-IgD syndrome (OMIM no. 260920) presenting with cutaneous lesions, recurrent fever, lymphadenopathy, hepatosplenomegaly, and arthralgia [161]. Hyper-IgD most frequently presents with erythematous macules and papules that do not consistently show features of aberrant keratinization in histopathology [162].

#### Treatment

Porokeratosis usually runs a protracted and recalcitrant course [154]. To date, no causative treatment normalizing the mevalonate pathway has been elaborated. Common treatment modalities involve topical agents (steroids, retinoids, imiquimod, 5-fluorouracil) and systemic retinoids [154]. Procedures such as surgical excision, cryotherapy, and laser therapy can be considered. Recently, several reports showed promising results of combination creams containing cholesterol with simvastatin or lovastatin [160, 163–166].

### JAK1-Associated Autoinflammatory Keratinization Disease with Hepatitis and Autism

#### Clinical Features

Recently, a case report of a child with early-onset ichthyotic eczema coexisting with eosinophilia, liver abnormalities, growth retardation, and autism was published [92]. Quickly progressing hepatic cirrhosis produced the necessity to perform liver transplantation at the age of 3 years. Furthermore, the patient presented growth retardation, moderate motor impairment, learning disability, and hyperlipidemia. Similar cutaneous findings were also reported in other studies analyzing the role of increased JAK1 activation, underlying a novel potential pathway of AiKDs with associated systemic symptoms [167–169].

#### Histology

Skin pathology showed compact acanthosis, hyperkeratosis spongiosis, and superficial lymphocytic infiltrate [92]. Hepatic biopsy revealed features of cirrhosis.

#### Genetic Background

A case of JAK1-associated disorder first denoted as an autoinflammatory keratinization disease was described by Takeichi et al. and was due to a de novo heterozygous c.1786C > G mutation in the *JAK1* gene [92]. As mentioned above, the patient also showed eosinophilia and hepatic and central nervous system involvement. Five years earlier, a family with heterozygous mutation in c.1901C > A *JAK1* variant presenting widespread lesions reminding atopic dermatitis, pronounced eosinophilia, eosinophilic infiltration of the liver and gastrointestinal tract, and liver cysts had been described (OMIM no. 618999) [169]. Strikingly similar symptoms were also reported in another patient described by Gruber et al. who harbored a *de novo* heterozygous c.2108 G > T *JAK1* mutation [168]. Therefore, it seems that regardless of the particular variant, *JAK1*-activating mutations result in superficial inflammatory lesions and eosinophilia associated with internal organ involvement (particularly the gastrointestinal tract).

#### Treatment

The patient described by Takeichi et al. received no targeted treatment and died of an unknown cause at the age of 22 years [92]. Based on the experimental data, it is known that increased JAK1 activity results in the upregulation of the TNF, IFN-γ, and IL-6 signaling pathways. Indeed, Del Bel et al. reported favorable treatment outcomes of ruxolitinib [169]. Therefore, selective JAK1 inhibitors (e.g., upadacitinib) and anti-TNF biologics could prove successful in the treatment of similar autoinflammatory syndromes [92].

### Keratosis Linearis with Ichthyosis Congenita and Sclerosing Keratoderma Syndrome

#### Clinical Features

Keratosis linearis with ichthyosis congenita and sclerosing keratoderma (KLICK) syndrome (OMIM no. 601952) is an extremely rare autosomal recessive skin disorder. Patients present palmoplantar keratoderma, hyperkeratotic plaques (often in a linear distribution), ichthyosiform scaling, circular constrictions around the fingers, and multiple linear papular lesions in the arm folds and on the wrists [94]. Additionally, an atypical case of KLICK has been recently published [170]. The patient presented widespread erythematous lesions that were well demarcated and nonmigratory as well as diffuse thin white scaling.

#### Histology

Cutaneous biopsy revealed irregular hyperplasia, hypergranulosis, superficial hyperkeratosis, parakeratosis, and superficial lymphohistiocytic infiltrates [94].

#### Genetic Background

To date, all cases of KLICK syndrome have been linked to homozygous 1-bp deletion in the 5′ untranslated region of the *POMP* gene [94]. *POMP* encodes a proteasome maturation protein whose downregulation likely results in the accumulation of undegraded ubiquitinylated proteins, thereby leading to cellular stress and abnormal keratinization. So far, it remains unknown as to why one of the cases described by Onnis et al. [170] presented with atypical erythematous lesions reminiscent of erythrokeratoderma.

#### Treatment

Based on the scarce data, it seems that treatment with retinoids, mainly acitretin or etretinate, could alleviate the symptoms of KLICK [94].

### Epidermal Growth Factor Receptor Deficiency

#### Clinical Features

EGFR deficiency (OMIM no. 131550) was first described in 2014 as a cause of papulo-pustular rash, loss of scalp hair, and trichomegaly in a premature infant of Polish-Roma origin. The child also suffered from gastrointestinal and respiratory symptoms (watery diarrhea, respiratory difficulties, and bronchiolitis) and died of infection and electrolyte imbalance at the age of 2.5 years [171]. The same etiology was demonstrated in two siblings with skin thinning, dryness, ichthyotic lesions, absence of subcutaneous fat, and alopecia of the scalp and eyebrows [172]. The children also showed severe gastrointestinal and respiratory abnormalities and died in early infancy. These reports were followed by a series of 18 EGFR deficiency cases presenting similar clinical features [98]. At the time of publication, only one patient survived until adolescence. Cutaneous symptoms were described as ichthyosiform dermatitis with hyperkeratinization and formation of chronic papules and pustules.

EGFR deficiency should be differentiated with neonatal inflammatory skin and bowel disease type 1 resulting from homozygous mutations of *ADAM17* [173, 174]*.* This condition also presents with neonatal-onset psoriasiform erythroderma, diarrhea, short or broken hair, and wiry or disorganized eyelashes. Clinical similarities between EGFR deficiency and neonatal inflammatory skin and bowel disease type 1 could be attributed to the fact that ADAM17 converts several molecules, including EGF, TNF, and transforming growth factor α (TGF-α). Therefore, described symptoms should prompt genetic screening for both *ADAM17* and *EGFR.*

#### Histology

Cutaneous biopsies performed in several confirmed EGFR cases revealed diffuse parakeratosis, mild acanthosis, superficial perivascular and perifollicular lymphocytic infiltrate, dermal atrophy, and wiry-appearing collagen fibers [98, 171, 172].

#### Genetic Background

EGFR deficiency has been linked to homozygous (c.1283G > A) or compound heterozygous (c.292C > T and c.1094 T > A) mutations in the *EGFR* gene [98]. The former has been described in patients of Roman origin, while the latter has been reported only in one case from Japan. All patients with EGFR deficiency presented strikingly similar symptoms, and all but one died in early infancy. It seems that both genotypes lead to the onset of severe cutaneous lesions and alopecia frequently associated with gastrointestinal and renal symptoms.

#### Treatment

The treatment of EGFR deficiency is symptomatic and involves fluid therapy, electrolyte supplementation, antiseptics, emollients, and photoprotection [98, 171, 172]. The patients usually die in early childhood due to infectious complications, gastrointestinal tract perforation, or respiratory distress.

## Conclusions

Autoinflammatory keratinization diseases are a challenging group of disorders manifesting hyperkeratotic inflammatory lesions. Innate immunity plays a central role in the pathogenesis of these entities. Understanding the mechanisms leading to a sporadic onset of superficial dermal and epidermal inflammation altering the keratinization process is key for efficient differentiation and treatment. Family history, early onset, and presence of systemic syndromes are suggestive features that should prompt genetic screening. The growing knowledge of the molecular pathways affecting the activation of the innate immune system will probably help to identify new subgroups of autoinflammatory keratinization diseases in the future.

## References

[1] French FMF Consortium (1997). A candidate gene for familial Mediterranean fever. Nat Genet.

[2] Havnaer A, Han G (2019). Autoinflammatory disorders: a review and update on pathogenesis and treatment. Am J Clin Dermatol.

[3] Broderick L, Hoffman HM (2022). IL-1 and autoinflammatory disease: biology, pathogenesis and therapeutic targeting. Nat Rev Rheumatol.

[4] McDermott MF, Aksentijevich I, Galon J (1999). Germline mutations in the extracellular domains of the 55 kDa TNF receptor, TNFR1, define a family of dominantly inherited autoinflammatory syndromes. Cell.

[5] Akiyama M, Takeichi T, McGrath JA, Sugiura K (2017). Autoinflammatory keratinization diseases. J Allergy Clin Immunol.

[6] Manthiram K, Zhou Q, Aksentijevich I, Kastner DL (2017). The monogenic autoinflammatory diseases define new pathways in human innate immunity and inflammation. Nat Immunol.

[7] Demaria O, Cornen S, Daëron M, Morel Y, Medzhitov R, Vivier E (2019). Harnessing innate immunity in cancer therapy. Nature.

[8] Paludan SR, Pradeu T, Masters SL, Mogensen TH (2021). Constitutive immune mechanisms: mediators of host defence and immune regulation. Nat Rev Immunol.

[9] Li D, Wu M (2021). Pattern recognition receptors in health and diseases. Signal Transduct Target Ther.

[10] Hillion S, Arleevskaya MI, Blanco P (2020). The innate part of the adaptive immune system. Clin Rev Allergy Immunol.

[11] de Jesus AA, Canna SW, Liu Y, Goldbach-Mansky R (2015). Molecular mechanisms in genetically defined autoinflammatory diseases: disorders of amplified danger signaling. Annu Rev Immunol.

[12] Nigrovic PA, Lee PY, Hoffman HM (2020). Monogenic autoinflammatory disorders: conceptual overview, phenotype, and clinical approach. J Allergy Clin Immunol.

[13] Meier-Schiesser B, French LE (2021). Autoinflammatory syndromes. J Dtsch Dermatol Ges.

[14] Behzadi P, Sameer AS, Nissar S (2022). The interleukin-1 (IL-1) superfamily cytokines and their single nucleotide polymorphisms (SNPs). J Immunol Res.

[15] Hoffman HM, Broderick L (2016). The role of the inflammasome in patients with autoinflammatory diseases. J Allergy Clin Immunol.

[16] Hausmann A, Böck D, Geiser P (2020). Intestinal epithelial NAIP/NLRC4 restricts systemic dissemination of the adapted pathogen Salmonella typhimurium due to site-specific bacterial PAMP expression. Mucosal Immunol.

[17] Tuladhar S, Kanneganti TD (2020). NLRP12 in innate immunity and inflammation. Mol Aspects Med.

[18] Zhong FL, Mamaï O, Sborgi L (2016). Germline NLRP1 mutations cause skin inflammatory and cancer susceptibility syndromes via inflammasome activation. Cell.

[19] Yuan ZC, Xu WD, Liu XY, Liu XY, Huang AF, Su LC (2019). Biology of IL-36 signaling and its role in systemic inflammatory diseases. Front Immunol.

[20] Walter MR (2020). The role of structure in the biology of interferon signaling. Front Immunol.

[21] Lee AJ, Ashkar AA (2018). The dual nature of type I and type II interferons. Front Immunol.

[22] Platanias LC (2005). Mechanisms of type-I- and type-II-interferon-mediated signalling. Nat Rev Immunol.

[23] Wack A, Terczyńska-Dyla E, Hartmann R (2015). Guarding the frontiers: the biology of type III interferons. Nat Immunol.

[24] Mesev EV, LeDesma RA, Ploss A (2019). Decoding type I and III interferon signalling during viral infection. Nat Microbiol.

[25] Crow YJ, Stetson DB (2022). The type I interferonopathies: 10 years on. Nat Rev Immunol.

[26] Lee-Kirsch MA (2017). The type I interferonopathies. Annu Rev Med.

[27] Liu T, Zhang L, Joo D, Sun SC (2017). NF-κB signaling in inflammation. Signal Transduct Target Ther.

[28] Taniguchi K, Karin M (2018). NF-κB, inflammation, immunity and cancer: coming of age. Nat Rev Immunol.

[29] Bertin J, Wang L, Guo Y (2001). CARD11 and CARD14 are novel caspase recruitment domain (CARD)/membrane-associated guanylate kinase (MAGUK) family members that interact with BCL10 and activate NF-kappa B. J Biol Chem.

[30] Israel L, Mellett M (2018). Clinical and genetic heterogeneity of CARD14 mutations in psoriatic skin disease. Front Immunol.

[31] Aksentijevich I, Zhou Q (2017). NF-κB pathway in autoinflammatory diseases: dysregulation of protein modifications by ubiquitin defines a new category of autoinflammatory diseases. Front Immunol.

[32] Milner JD (2015). PLAID: a syndrome of complex patterns of disease and unique phenotypes. J Clin Immunol.

[33] Gernez Y, de Jesus AA, Alsaleem H (2019). Severe autoinflammation in 4 patients with C-terminal variants in cell division control protein 42 homolog (CDC42) successfully treated with IL-1β inhibition. J Allergy Clin Immunol.

[34] Vece TJ, Watkin LB, Nicholas SK (2016). Copa syndrome: a novel autosomal dominant immune dysregulatory disease. J Clin Immunol.

[35] Doria A, Zen M, Bettio S (2012). Autoinflammation and autoimmunity: bridging the divide. Autoimmun Rev.

[36] Szekanecz Z, McInnes IB, Schett G, Szamosi S, Benkő S, Szűcs G (2021). Autoinflammation and autoimmunity across rheumatic and musculoskeletal diseases. Nat Rev Rheumatol.

[37] Kumar V (2019). A STING to inflammation and autoimmunity. J Leukoc Biol.

[38] Shaw PJ, McDermott MF, Kanneganti TD (2011). Inflammasomes and autoimmunity. Trends Mol Med.

[39] Tartey S, Kanneganti T (2020). Inflammasomes in the pathophysiology of autoinflammatory syndromes. J Leukoc Biol.

[40] Evavold CL, Kagan JC (2018). How inflammasomes inform adaptive immunity. J Mol Biol.

[41] Martynova E, Rizvanov A, Urbanowicz RA, Khaiboullina S (2022). Inflammasome contribution to the activation of Th1, Th2, and Th17 immune responses. Front Microbiol.

[42] Hagberg N, Berggren O, Leonard D et al (2011) IFN-α production by plasmacytoid dendritic cells stimulated with RNA-containing immune complexes is promoted by NK cells via MIP-1β and LFA-1. J Immunol (Balt Md 1950) 186(9):5085–5094. 10.4049/jimmunol.1003349 10.4049/jimmunol.1003349 21430220

[43] Panda SK, Kolbeck R, Sanjuan MA (2017). Plasmacytoid dendritic cells in autoimmunity. Curr Opin Immunol.

[44] Negroni A, Pierdomenico M, Cucchiara S, Stronati L (2018). NOD2 and inflammation: current insights. J Inflamm Res.

[45] Khunsriraksakul C, Markus H, Olsen NJ, Carrel L, Jiang B, Liu DJ (2022). Construction and application of polygenic risk scores in autoimmune diseases. Front Immunol.

[46] Ramos PS, Shedlock AM, Langefeld CD (2015). Genetics of autoimmune diseases: insights from population genetics. J Hum Genet.

[47] Griffiths CEM, Armstrong AW, Gudjonsson JE, Barker JNWN (2021). Psoriasis. Lancet.

[48] Akiyama M, Takeichi T, McGrath JA, Sugiura K (2018). Autoinflammatory keratinization diseases: an emerging concept encompassing various inflammatory keratinization disorders of the skin. J Dermatol Sci.

[49] Akiyama M (2020). Autoinflammatory keratinization diseases (AiKDs): expansion of disorders to be included. Front Immunol.

[50] Craiglow BG, Boyden LM, Hu R (2018). CARD14-associated papulosquamous eruption: a spectrum including features of psoriasis and pityriasis rubra pilaris. J Am Acad Dermatol.

[51] Frew JW (2020). Hidradenitis suppurativa is an autoinflammatory keratinization disease: a review of the clinical, histologic, and molecular evidence. JAAD Int.

[52] Nomura T (2020). Hidradenitis Suppurativa as a potential subtype of autoinflammatory keratinization disease. Front Immunol.

[53] Gabay C, Towne JE (2015). Regulation and function of interleukin-36 cytokines in homeostasis and pathological conditions. J Leukoc Biol.

[54] Queen D, Ediriweera C, Liu L (2019). Function and regulation of IL-36 signaling in inflammatory diseases and cancer development. Front Cell Dev Biol.

[55] Buhl AL, Wenzel J (2019). Interleukin-36 in infectious and inflammatory skin diseases. Front Immunol.

[56] Henry CM, Sullivan GP, Clancy DM, Afonina IS, Kulms D, Martin SJ (2016). Neutrophil-derived proteases escalate inflammation through activation of IL-36 family cytokines. Cell Rep.

[57] Murrieta-Coxca J, Rodríguez-Martínez S, Cancino-Diaz M, Markert U, Favaro R, Morales-Prieto D (2019). IL-36 cytokines: regulators of inflammatory responses and their emerging role in immunology of reproduction. Int J Mol Sci.

[58] Macleod T, Berekmeri A, Bridgewood C, Stacey M, McGonagle D, Wittmann M (2021). The immunological impact of IL-1 family cytokines on the epidermal barrier. Front Immunol.

[59] Saito K, Iwata Y, Fukushima H (2020). IL-36 receptor antagonist deficiency resulted in delayed wound healing due to excessive recruitment of immune cells. Sci Rep.

[60] Marrakchi S, Guigue P, Renshaw BR (2011). Interleukin-36–receptor antagonist deficiency and generalized pustular psoriasis. N Engl J Med.

[61] Onoufriadis A, Simpson MA, Pink AE (2011). Mutations in IL36RN/IL1F5 are associated with the severe episodic inflammatory skin disease known as generalized pustular psoriasis. Am J Hum Genet.

[62] Sugiura K, Takeichi T, Kono M et al (2012) A novel IL36RN/IL1F5 homozygous nonsense mutation, p.Arg10X, in a Japanese patient with adult-onset generalized pustular psoriasis. Br J Dermatol 167(3):699–701. 10.1111/j.1365-2133.2012.10953.x 10.1111/j.1365-2133.2012.10953.x 22428995

[63] Sugiura K, Takemoto A, Yamaguchi M (2013). The majority of generalized pustular psoriasis without psoriasis vulgaris is caused by deficiency of interleukin-36 receptor antagonist. J Invest Dermatol.

[64] Abbas O, Itani S, Ghosn S (2013). Acrodermatitis continua of Hallopeau is a clinical phenotype of DITRA: evidence that it is a variant of pustular psoriasis. Dermatology.

[65] Sugiura K, Oiso N, Iinuma S (2014). IL36RN mutations underlie impetigo herpetiformis. J Invest Dermatol.

[66] Sugiura K (2022). Role of interleukin 36 in generalised pustular psoriasis and beyond. Dermatol Ther.

[67] Marzano AV, Damiani G, Genovese G, Gattorno M (2018) A dermatologic perspective on autoinflammatory diseases. Clin Exp Rheumatol 36 Suppl 110(1):32–38 29742056

[68] Cowen EW (2012). DIRA, DITRA, and new insights into pathways of skin inflammation: what’s in a name?. Arch Dermatol.

[69] Hannigan GD, Grice EA (2013). Microbial ecology of the skin in the era of metagenomics and molecular microbiology. Cold Spring Harb Perspect Med.

[70] Johnston A, Xing X, Wolterink L (2017). IL-1 and IL-36 are dominant cytokines in generalized pustular psoriasis. J Allergy Clin Immunol.

[71] Fenini G, Karakaya T, Hennig P, Di Filippo M, Slaufova M, Beer HD (2022). NLRP1 inflammasome activation in keratinocytes: increasing evidence of important roles in inflammatory skin diseases and immunity. J Invest Dermatol.

[72] Burian M, Yazdi AS (2018). NLRP1 is the key inflammasome in primary human keratinocytes. J Invest Dermatol.

[73] Mitchell PS, Sandstrom A, Vance RE (2019). The NLRP1 inflammasome: new mechanistic insights and unresolved mysteries. Curr Opin Immunol.

[74] Drutman SB, Haerynck F, Zhong FL (2019). Homozygous NLRP1 gain-of-function mutation in siblings with a syndromic form of recurrent respiratory papillomatosis. Proc Natl Acad Sci USA.

[75] Herlin T, Jørgensen SE, Høst C (2020). Autoinflammatory disease with corneal and mucosal dyskeratosis caused by a novel NLRP1 variant. Rheumatology.

[76] Li M, Lay K, Zimmer A et al (2023) A homozygous p.Leu813Pro gain-of-function NLRP1 variant causes phenotypes of different severity in two siblings. Br J Dermatol 188(2):259–267. 10.1093/bjd/ljac039 10.1093/bjd/ljac039 36763876

[77] Marrakchi S, Puig L (2022). Pathophysiology of generalized pustular psoriasis. Am J Clin Dermatol.

[78] Fuchs-Telem D, Sarig O, van Steensel MAM (2012). Familial pityriasis rubra pilaris is caused by mutations in CARD14. Am J Hum Genet.

[79] Wang M, Zhang S, Zheng G (2018). Gain-of-function mutation of Card14 leads to spontaneous psoriasis-like skin inflammation through enhanced keratinocyte response to IL-17A. Immunity.

[80] Mellett M, Meier B, Mohanan D (2018). CARD14 gain-of-function mutation alone is sufficient to drive IL-23/IL-17–mediated psoriasiform skin inflammation in vivo. J Invest Dermatol.

[81] Setta-Kaffetzi N, Simpson MA, Navarini AA (2014). AP1S3 mutations are associated with pustular psoriasis and impaired toll-like receptor 3 trafficking. Am J Hum Genet.

[82] Mahil SK, Twelves S, Farkas K (2016). AP1S3 mutations cause skin autoinflammation by disrupting keratinocyte autophagy and up-regulating IL-36 production. J Invest Dermatol.

[83] Mössner R, Wilsmann-Theis D, Oji V (2018). The genetic basis for most patients with pustular skin disease remains elusive. Br J Dermatol.

[84] Twelves S, Mostafa A, Dand N (2019). Clinical and genetic differences between pustular psoriasis subtypes. J Allergy Clin Immunol.

[85] Liao P, Hemmerlin A, Bach TJ, Chye ML (2016). The potential of the mevalonate pathway for enhanced isoprenoid production. Biotechnol Adv.

[86] Chang WC, Song H, Liu HW, Liu P (2013) Current development in isoprenoid precursor biosynthesis and regulation. Curr Opin Chem Biol 17(4):571–579. 10.1016/j.cbpa.2013.06.020 10.1016/j.cbpa.2013.06.020 PMC4068245 23891475

[87] Takeichi T, Akiyama M (2019). Familial or sporadic porokeratosis as an autoinflammatory keratinization disease. J Dermatol.

[88] Pontillo A, Paoluzzi E, Crovella S (2010). The inhibition of mevalonate pathway induces upregulation of NALP3 expression: new insight in the pathogenesis of mevalonate kinase deficiency. Eur J Hum Genet.

[89] Hu X, li J, Fu M, Zhao X, Wang W (2021) The JAK/STAT signaling pathway: from bench to clinic. Signal Transduct Target Ther 6(1):402. 10.1038/s41392-021-00791-1 10.1038/s41392-021-00791-1 PMC8617206 34824210

[90] Choy EH (2019). Clinical significance of Janus Kinase inhibitor selectivity. Rheumatol Oxf Engl.

[91] Majoros A, Platanitis E, Kernbauer-Hölzl E, Rosebrock F, Müller M, Decker T (2017) Canonical and non-canonical aspects of JAK–STAT signaling: lessons from interferons for cytokine responses. Front Immunol 8. 10.3389/fimmu.2017.00029 10.3389/fimmu.2017.00029 PMC5266721 28184222

[92] Takeichi T, Lee JYW, Okuno Y (2022). Autoinflammatory keratinization disease with hepatitis and autism reveals roles for JAK1 kinase hyperactivity in autoinflammation. Front Immunol.

[93] Poli MC, Ebstein F, Nicholas SK (2018). Heterozygous truncating variants in POMP escape nonsense-mediated decay and cause a unique immune dysregulatory syndrome. Am J Hum Genet.

[94] Takeichi T, Akiyama M (2020). KLICK syndrome linked to a POMP mutation has features suggestive of an autoinflammatory keratinization disease. Front Immunol.

[95] Park K, Lee SE, Shin KO, Uchida Y (2019). Insights into the role of endoplasmic reticulum stress in skin function and associated diseases. FEBS J.

[96] Tran QT, Kennedy LH, Leon Carrion S (2012). EGFR regulation of epidermal barrier function. Physiol Genomics.

[97] Takeichi T, Akiyama M (2021) Systemic inflammatory diseases due to germ line EGFR mutations, with features suggestive of autoinflammatory keratinization diseases. J Dermatol 48(1). 10.1111/1346-8138.15612 10.1111/1346-8138.15612 32940378

[98] Mazurova S, Tesarova M, Zeman J (2020). Fatal neonatal nephrocutaneous syndrome in 18 Roma children with *EGFR* deficiency. J Dermatol.

[99] Kanazawa N (2020). Designation of autoinflammatory skin manifestations with specific genetic backgrounds. Front Immunol.

[100] Hüffmeier U, Wätzold M, Mohr J, Schön MP, Mössner R (2014). Successful therapy with anakinra in a patient with generalized pustular psoriasis carrying *IL36RN* mutations. Br J Dermatol.

[101] Uppala R, Tsoi LC, Harms PW (2021). “Autoinflammatory psoriasis”—genetics and biology of pustular psoriasis. Cell Mol Immunol.

[102] Genovese G, Moltrasio C, Cassano N, Maronese CA, Vena GA, Marzano AV (2021). Pustular psoriasis: from pathophysiology to treatment. Biomedicines.

[103] Liu J, Ali K, Lou H, Wang L, Wu L (2022). First-trimester impetigo herpetiformis leads to stillbirth: a case report. Dermatol Ther.

[104] Misiak-Galazka M, Zozula J, Rudnicka L (2020). Palmoplantar pustulosis: recent advances in etiopathogenesis and emerging treatments. Am J Clin Dermatol.

[105] Mrowietz U, van de Kerkhof PCM (2011). Management of palmoplantar pustulosis: do we need to change? Management of palmoplantar pustulosis. Br J Dermatol.

[106] Hiraiwa T, Yamamoto T (2017). Nail involvement associated with palmoplantar pustulosis. Int J Dermatol.

[107] Bachelez H (2020). Pustular psoriasis: the dawn of a new era. Acta Derm Venereol.

[108] Choon SE, Navarini AA, Pinter A (2022). Clinical course and characteristics of generalized pustular psoriasis. Am J Clin Dermatol.

[109] Feldmeyer L, Heidemeyer K, Yawalkar N (2016). Acute generalized exanthematous pustulosis: pathogenesis, genetic background, clinical variants and therapy. Int J Mol Sci.

[110] Watts PJ, Khachemoune A (2016). Subcorneal pustular dermatosis: a review of 30 years of progress. Am J Clin Dermatol.

[111] Kocak M, Birol A, Erkek E, Bozdogan O, Atasoy P (2003). Juvenile subcorneal pustular dermatosis: a case report. Pediatr Dermatol.

[112] Johnson SA, Cripps DJ (1974). Subcorneal pustular dermatosis in children. Arch Dermatol.

[113] Razera F, Olm GS, Bonamigo RR (2011). Dermatoses neutrofílicas: parte II. An Bras Dermatol.

[114] Yoshikawa M, Rokunohe D, Kimura A (2021). Significance of *IL36RN* mutation analyses in the management of impetigo herpetiformis: a case report and review of published cases. J Dermatol.

[115] Kinoshita M, Ogawa Y, Takeichi T (2018). Impetigo herpetiformis with IL-36RN mutation successfully treated with secukinumab. Eur J Dermatol.

[116] Kanatani Y, Shinkuma S, Matsumoto Y et al (2022) Recurrence of impetigo herpetiformis carrying compound heterozygous mutations in IL36RN after remission with secukinumab. J Dermatol 49(3). 10.1111/1346-8138.16247 10.1111/1346-8138.16247 34806229

[117] Chhabra G, Chanana C, Verma P, Saxena A (2019) Impetigo herpetiformis responsive to secukinumab. Dermatol Ther 32(5). 10.1111/dth.13040 10.1111/dth.13040 31361940

[118] Navarini AA, Valeyrie-Allanore L, Setta-Kaffetzi N (2013). Rare variations in IL36RN in severe adverse drug reactions manifesting as acute generalized exanthematous pustulosis. J Invest Dermatol.

[119] Hospach T, Glowatzki F, Blankenburg F (2019). Scoping review of biological treatment of deficiency of interleukin-36 receptor antagonist (DITRA) in children and adolescents. Pediatr Rheumatol.

[120] Mansouri B, Benjegerdes K, Hyde K, Kivelevitch D (2016). Pustular psoriasis: pathophysiology and current treatment perspectives. Psoriasis Targets Ther.

[121] Mössner R, Thaci D, Mohr J (2008). Manifestation of palmoplantar pustulosis during or after infliximab therapy for plaque-type psoriasis: report on five cases. Arch Dermatol Res.

[122] Baum P, Visvanathan S, Garcet S (2022). Pustular psoriasis: molecular pathways and effects of spesolimab in generalized pustular psoriasis. J Allergy Clin Immunol.

[123] Federal Drug Administration. New drug approvals for 2022. Available at: https://www.accessdata.fda.gov/drugsatfda_docs/label/2022/761244s000lbl.pdf. Accessed 19 Jan 2023

[124] European Commission approves SPEVIGO^®^ (spesolimab) for generalized pustular psoriasis flares. Available at: https://www.boehringer-ingelheim.com/human-health/skin-diseases/gpp/european-commission-approves-spevigo-spesolimab-generalized. Accessed 19 Jan 2023

[125] Iznardo H, Puig L (2022). IL-1 family cytokines in inflammatory dermatoses: pathogenetic role and potential therapeutic implications. Int J Mol Sci.

[126] Fujita H, Terui T, Hayama K (2018). Japanese guidelines for the management and treatment of generalized pustular psoriasis: the new pathogenesis and treatment of GPP. J Dermatol.

[127] Mendonca LO, Malle L, Donovan FX (2017). Deficiency of interleukin-1 receptor antagonist (DIRA): report of the first Indian patient and a novel deletion affecting IL1RN. J Clin Immunol.

[128] Kolivras A, Meiers I, Sass U, Thompson CT (2021). Histologic patterns and clues to autoinflammatory diseases in children: what a cutaneous biopsy can tell us. Dermatopathology.

[129] Aksentijevich I, Masters SL, Ferguson PJ (2009). An autoinflammatory disease with deficiency of the interleukin-1–receptor antagonist. N Engl J Med.

[130] Jesus AA, Goldbach-Mansky R (2014). IL-1 blockade in autoinflammatory syndromes. Annu Rev Med.

[131] Dinarello CA, van der Meer JWM (2013). Treating inflammation by blocking interleukin-1 in humans. Semin Immunol.

[132] Gómez-García F, Sanz-Cabanillas JL, Viguera-Guerra I, Isla-Tejera B, Nieto AVG, Ruano J (2018). Scoping review on use of drugs targeting interleukin 1 pathway in DIRA and DITRA. Dermatol Ther.

[133] Grandemange S, Sanchez E, Louis-Plence P (2017). A new autoinflammatory and autoimmune syndrome associated with NLRP1 mutations: NAIAD (NLRP1-associated autoinflammation with arthritis and dyskeratosis). Ann Rheum Dis.

[134] From E, Philipsen HP, Thormann J (1978) Dyskeratosis benigna intraepithelialis mucosae et cutis hereditaria. A report of this disorder in father and son. J Cutan Pathol 5(3):105–115. 10.1111/j.1600-0560.1978.tb00947.x 10.1111/j.1600-0560.1978.tb00947.x 681574

[135] Soler VJ, Tran-Viet KN, Galiacy SD (2013). Whole exome sequencing identifies a mutation for a novel form of corneal intraepithelial dyskeratosis. J Med Genet.

[136] Mamaï O, Boussofara L, Denguezli M (2015). Multiple self-healing palmoplantar carcinoma: a familial predisposition to skin cancer with primary palmoplantar and conjunctival lesions. J Invest Dermatol.

[137] Böer A (2006). Keratosis lichenoides chronica: proposal of a concept: Am J Dermatopathol.

[138] Allingham RR, Seo B, Rampersaud E (2001). A duplication in chromosome 4q35 is associated with hereditary benign intraepithelial dyskeratosis. Am J Hum Genet.

[139] Yanoff M (1968). Hereditary benign intraepithelial dyskeratosis. Arch Ophthalmol.

[140] Seely M, Jackson K, Meeker A, Daluvoy M (2022). Case series of patients with hereditary benign intraepithelial dyskeratosis. Cornea.

[141] Bui T, Young JW, Frausto RF, Markello TC, Glasgow BJ, Aldave AJ (2016). hereditary benign intraepithelial dyskeratosis: report of a case and re-examination of the evidence for locus heterogeneity. Ophthalmic Genet.

[142] Venkatesan NN, Pine HS, Underbrink MP (2012). Recurrent respiratory papillomatosis. Otolaryngol Clin North Am.

[143] Cummings TJ, Dodd LG, Eedes CR, Klintworth GK (2008). Hereditary benign intraepithelial dyskeratosis: an evaluation of diagnostic cytology. Arch Pathol Lab Med.

[144] Agostini M, Valiati R, León JE, Romañach MJ, Scully C, de Almeida OP (2012). Mucocutaneous dyskeratosis with periodontal destruction and premature tooth loss. Oral Surg Oral Med Oral Pathol Oral Radiol.

[145] Patel A, Orban N (2021). Infantile recurrent respiratory papillomatosis: review of adjuvant therapies. J Laryngol Otol.

[146] Wang D, Chong VCL, Chong WS, Oon HH (2018). A review on pityriasis rubra pilaris. Am J Clin Dermatol.

[147] Roenneberg S, Biedermann T (2018). Pityriasis rubra pilaris: algorithms for diagnosis and treatment. J Eur Acad Dermatol Venereol.

[148] Magro CM, Crowson AN (1997). The clinical and histomorphological features of pityriasis rubra pilaris: a comparative analysis with psoriasis. J Cutan Pathol.

[149] Seitz CS, Freiberg RA, Hinata K, Khavari PA (2000). NF-kappaB determines localization and features of cell death in epidermis. J Clin Invest.

[150] Frare CP, Blumstein AJ, Paller AS (2021). *CARD14* -associated papulosquamous eruption (CAPE) in pediatric patients: three additional cases and review of the literature. Pediatr Dermatol.

[151] Hayden MS, Ghosh S (2014). Regulation of NF-κB by TNF family cytokines. Semin Immunol.

[152] Klein B, Treudler R, Dumann K (2022). Clinical response of CARD14-associated papulosquamous eruption to an anti-interleukin-17A antibody. Br J Dermatol.

[153] Chiramel MJ, Sathishkumar D, Edison ES, George R (2020). Two cases of *CARD14* -associated papulosquamous eruption from India. Pediatr Dermatol.

[154] Das A, Vasudevan B, Talwar A (2021) Porokeratosis: an enigma beginning to unravel. Indian J Dermatol Venereol Leprol 88:291–299. 10.25259/IJDVL_806_20 10.25259/IJDVL_806_20 34877845

[155] Löhrer R, Neumann-Acikel A, Eming R (2010). A case of linear porokeratosis superimposed on disseminated superficial actinic porokeratosis. Case Rep Dermatol.

[156] Niimi Y, Kawana S (2009). Type 2 segmental manifestation of disseminated superficial actinic porokeratosis in a 7-year-old girl. Eur J Dermatol.

[157] Sommerlad M, Lock A, Moir G (2016). Linear porokeratosis with multiple squamous cell carcinomas successfully treated by electrochemotherapy. Br J Dermatol.

[158] Leng Y, Yan L, Feng H (2018). Mutations in mevalonate pathway genes in patients with familial or sporadic porokeratosis. J Dermatol.

[159] Touitou I (2022). Twists and turns of the genetic story of mevalonate kinase-associated diseases: a review. Genes Dis.

[160] Atzmony L, Choate KA (2019). Second-hit somatic mutations in mevalonate pathway genes underlie porokeratosis. J Invest Dermatol.

[161] Mulders-Manders CM, Simon A (2015). Hyper-IgD syndrome/mevalonate kinase deficiency: what is new?. Semin Immunopathol.

[162] Drenth JPH (1994). Cutaneous manifestations and histologic findings in the hyperimmunoglobulinemia D syndrome. Arch Dermatol.

[163] Byth LA, Byth J (2021). Topical simvastatin–cholesterol for disseminated superficial actinic porokeratosis: an open-label, split-body clinical trial. Australas J Dermatol.

[164] Diep D, Janitz T, Kannan KS, et al. Bilateral linear porokeratosis treated with topical cholesterol 2%/lovastatin 2%. *Cureus*. Published online July 31, 2022. 10.7759/cureus.27540 10.7759/cureus.27540 PMC9428266 36060323

[165] Blue E, Abbott J, Bowen A, Cipriano SD (2021). Linear porokeratosis with bone abnormalities treated with compounded topical 2% cholesterol/2% lovastatin ointment. Pediatr Dermatol.

[166] Tomsitz D, Biedermann T (2022) Successful treatment of disseminated superficial actinic porokeratosis with topical 2% cholesterol/ 2% lovastatin cream: a case series with 7 patients. J Eur Acad Dermatol Venereol 36(1). 10.1111/jdv.17619 10.1111/jdv.17619 34418182

[167] Eletto D, Burns SO, Angulo I (2016). Biallelic JAK1 mutations in immunodeficient patient with mycobacterial infection. Nat Commun.

[168] Gruber CN, Calis JJA, Buta S (2020). Complex autoinflammatory syndrome unveils fundamental principles of JAK1 kinase transcriptional and biochemical function. Immunity.

[169] Del Bel KL, Ragotte RJ, Saferali A (2017). JAK1 gain-of-function causes an autosomal dominant immune dysregulatory and hypereosinophilic syndrome. J Allergy Clin Immunol.

[170] Onnis G, Bourrat E, Jonca N (2018). KLICK syndrome: an unusual phenotype. Br J Dermatol.

[171] Campbell P, Morton PE, Takeichi T (2014). Epithelial inflammation resulting from an inherited loss-of-function mutation in EGFR. J Invest Dermatol.

[172] Ganetzky R, Finn E, Bagchi A (2015). EGFR mutations cause a lethal syndrome of epithelial dysfunction with progeroid features. Mol Genet Genomic Med.

[173] Blaydon DC, Biancheri P, Di WL (2011). Inflammatory skin and bowel disease linked to *ADAM17* deletion. N Engl J Med.

[174] Samuelov L, Sarig O, Malovitski K (2022). Neonatal inflammatory skin and bowel disease type 1 caused by a complex genetic defect and responsive to combined anti-tumour necrosis factor-α and interleukin-12/23 blockade. Br J Dermatol.

